# Proteogenomics in cerebrospinal fluid and plasma reveals new biological fingerprint of cerebral small vessel disease

**DOI:** 10.1038/s43587-025-01006-w

**Published:** 2025-11-20

**Authors:** Ilana Caro, Daniel Western, Shinichi Namba, Na Sun, Shuji Kawaguchi, Yunye He, Masashi Fujita, Gennady Roshchupkin, Tim D’Aoust, Marie-Gabrielle Duperron, Muralidharan Sargurupremraj, Ami Tsuchida, Masaru Koido, Marziehsadat Ahmadi, Chengran Yang, Jigyasha Timsina, Laura Ibanez, Koichi Matsuda, Yutaka Suzuki, Yoshiya Oda, Akinori Kanai, Pouria Jandaghi, Markus Munter, Daniel Auld, Iana Astafeva, Raquel Puerta, Jerome I. Rotter, Bruce M. Psaty, Joshua C. Bis, WT Longstreth, Thierry Couffinhal, Pablo García-González, Vanesa Pytel, Marta Marquié, Amanda Cano, Mercè Boada, Marc Joliot, Mark Lathrop, Quentin Le Grand, Lenore J. Launer, Joanna M. Wardlaw, Myriam Heiman, Agustin Ruiz, Paul M. Matthews, Sudha Seshadri, Myriam Fornage, Hieab Adams, Aniket Mishra, David-Alexandre Trégouët, Yukinori Okada, Manolis Kellis, Philip L. De Jager, Christophe Tzourio, Yoichiro Kamatani, Fumihiko Matsuda, Carlos Cruchaga, Stéphanie Debette

**Affiliations:** 1 Bordeaux Population Health Research Center, Inserm U1219, Université de Bordeaux, Bordeaux, France; 2 Department of Psychiatry, Washington University School of Medicine, St Louis, MO USA; 3 NeuroGenomics and Informatics Center, Washington University School of Medicine, St Louis, MO USA; 4 Department of Genome Informatics, Graduate School of Medicine, The University of Tokyo, Tokyo, Japan; 5 Department of Statistical Genetics, Osaka University Graduate School of Medicine, Suita, Japan; 6 Laboratory for Systems Genetics, RIKEN Center for Integrative Medical Sciences, Suita, Japan; 7 MIT Computer Science and Artificial Intelligence Laboratory, Cambridge, MA USA; 8 Broad Institute of MIT and Harvard, Cambridge, MA USA; 9 Center for Genomic Medicine, Kyoto University Graduate School of Medicine, Kyoto, Japan; 10 Graduate School of Frontier Sciences, The University of Tokyo, Tokyo, Japan; 11 Department of Neurology, Center for Translational and Computational Neuroimmunology, Columbia University Irving Medical Center, New York, NY USA; 12 Department of Epidemiology, Erasmus MC University Medical Center Rotterdam, Rotterdam, the Netherlands; 13 Department of Neurology, Institute for Neurodegenerative Diseases, CHU de Bordeaux, Bordeaux, France; 14 Glenn Biggs Institute for Alzheimer’s and Neurodegenerative Diseases, University of Texas Health Sciences Center, San Antonio, TX USA; 15 Institut des Maladies Neurodégénératives, IMN-UMR5293, Université de Bordeaux, CNRS, CEA, Bordeaux, France; 16 Victor Phillip Dahdaleh Institute of Genomic Medicine, McGill University, Montreal, Quebec Canada; 17 Graduate School of Medicine, The University of Tokyo, Tokyo, Japan; 18 Ace Alzheimer Center Barcelona, Universitat Internacional de Catalunya, Barcelona, Spain; 19 The Institute for Translational Genomics and Population Sciences, Department of Pediatrics, The Lundquist Institute for Biomedical Innovation at Harbor-UCLA Medical Center, Torrance, CA USA; 20 Cardiovascular Health Research Unit, Department of Medicine, University of Washington, Seattle, WA USA; 21 Department of Epidemiology, University of Washington, Seattle, WA USA; 22 Department of Health Systems and Population Health, University of Washington, Seattle, WA USA; 23 Department of Neurology, University of Washington, Seattle, WA USA; 24 Inserm U1034, Biologie Des Maladies Cardiovasculaires, Université de Bordeaux, Bordeaux, France; 25 CIBERNED, Network Center for Biomedical Research in Neurodegenerative Diseases, National Institute of Health Carlos III, Madrid, Spain; 26 Population Health Sciences, German Center for Neurodegenerative Diseases (DZNE), Bonn, Germany; 27 Intramural Research Program, National Institue on Aging, Baltimore, MD USA; 28 Centre for Clinical Brain Sciences, University of Edinburgh, Chancellor’s Building, Edinburgh, UK; 29 UK Dementia Research Institute Centre at the University of Edinburgh and Centre for Vascular Dementia Research, Edinburgh, UK; 30 Department of Brain and Cognitive Sciences, Massachusetts Institute of Technology, Cambridge, MA USA; 31 The Picower Institute for Learning and Memory, Massachusetts Institute of Technology, Cambridge, MA USA; 32 Department of Brain Sciences, Imperial College, London, UK; 33 UK Dementia Research Institute Centre at Imperial, Imperial College, London, UK; 34 Brown Foundation Institute of Molecular Medicine, McGovern Medical School, University of Texas Health Science Center at Houston, Houston, TX USA; 35 Human Genetics Center, School of Public Health, University of Texas Health Science Center at Houston, Houston, TX USA; 36 Department of Human Genetics, Radboud University Medical Center, Nijmegen, the Netherlands; 37 Latin American Brain Health (BrainLat), Universidad Adolfo Ibáñez, Santiago, Chile; 38 Laboratory of Statistical Immunology, Immunology Frontier Research Center (WPI-IFReC), Osaka University, Suita, Japan; 39 Premium Research Institute for Human Metaverse Medicine (WPI-PRIMe), Osaka University, Suita, Japan; 40 Institut du Cerveau (ICM), Paris Brain Institute, INSERM U1127, UMR CNRS 7225 Paris, Sorbonne Université, Assistance Publique des Hôpitaux de Paris, Paris, France

**Keywords:** Cerebrovascular disorders, Neurovascular disorders

## Abstract

Cerebral small vessel disease (cSVD) is a leading cause of stroke and dementia with no specific treatment, of which molecular mechanisms remain poorly understood. To identify potential biomarkers and therapeutic targets, we applied Mendelian randomization to examine over 2,500 proteins measured in plasma and, uniquely, cerebrospinal fluid, in relation to magnetic resonance imaging (MRI) markers of cSVD in more than 40,000 individuals. Here we show that 49 proteins are associated with MRI markers of cSVD, most prominently in cerebrospinal fluid. We highlight associations that are consistent across platforms and ancestries, and supported by complementary observational analyses, and we explore differences between fluids. The proteins are enriched in pathways related to the extracellular matrix, immune response and microglial activity. Many also associate with stroke and dementia, and several correspond to existing drug targets. Together, these findings reveal a robust biological fingerprint of cSVD and highlight opportunities for biomarker and drug discovery and repositioning.

## Abstract (web summary)

By integrating large-scale genomic and proteomic data in cerebrospinal fluid and plasma, the authors identify 49 proteins linked to MRI markers of cerebral small vessel disease, highlighting extracellular matrix and immune pathways, with biomarker and therapeutic potential.

## Main

Characterized by changes in the structure and function of small brain vessels, cSVD is a leading cause of ischemic and hemorrhagic stroke, cognitive decline and dementia. cSVD is extremely common with increasing age and most often ‘covert’, namely detectable on brain magnetic resonance imaging (MRI) in the absence of clinical stroke. Covert cSVD is associated with changes in cognitive performance, gait, balance and mood disturbances and portends a considerably increased risk of developing stroke and dementia, thus representing a major target to prevent these disabling conditions and promote healthier brain aging^1^. The most common and heritable MRI markers of covert cerebral small vessel disease (MRI-cSVD) are white matter hyperintensities of presumed vascular origin (WMHs) and perivascular spaces (PVSs)^2^.

While hypertension is the strongest known risk factor for cSVD^1^, vascular risk factors explain only a limited fraction of MRI-cSVD variability in older age^3^, and drugs specifically targeting pathological processes underlying cSVD are lacking. Genomics can provide a strong foundation for mechanistic studies and drug target discovery^4^. Recent genetic studies identified >70 genetic risk loci for cSVD^5,6^, but causal genes and pathways remain poorly understood. As disease occurrence reflects the complex interplay of factors beyond DNA sequence, there is growing interest in identifying circulating biomarkers for clinical use, such as proteins, capturing these downstream factors, to unravel the underlying biology and accelerate omics-driven drug discovery^7^. While large-scale proteomic investigations have recently been conducted for stroke and dementia^7–13^, so far cSVD proteome studies were performed on limited sets of proteins, in small studies of European ancestry (*N* < 5,000)^14–18^, and only associations with plasma proteins were explored. We hypothesize that, while plasma may provide easy-access biomarkers, cerebrospinal fluid (CSF), the fluid circulating in PVSs, could reveal a more accurate biological fingerprint of cSVD.

We used two-sample Mendelian randomization (2SMR), leveraging large proteomic and genomic resources, to investigate the relation of circulating protein levels in CSF and plasma with WMHs and PVS burden. We then conducted a multipronged follow-up of identified associations in independent samples, across fluids, proteomic platforms, ancestries and the lifespan, using both 2SMR and individual-level data from observational studies. We also tested the relation of cSVD-associated proteins with risk of stroke and dementia, and deciphered cell types and pathways involved using single-cell sequencing resources. Finally, we combined our results with pharmacological databases for proteomics-driven drug discovery.

## Results

**Fig. 1:**
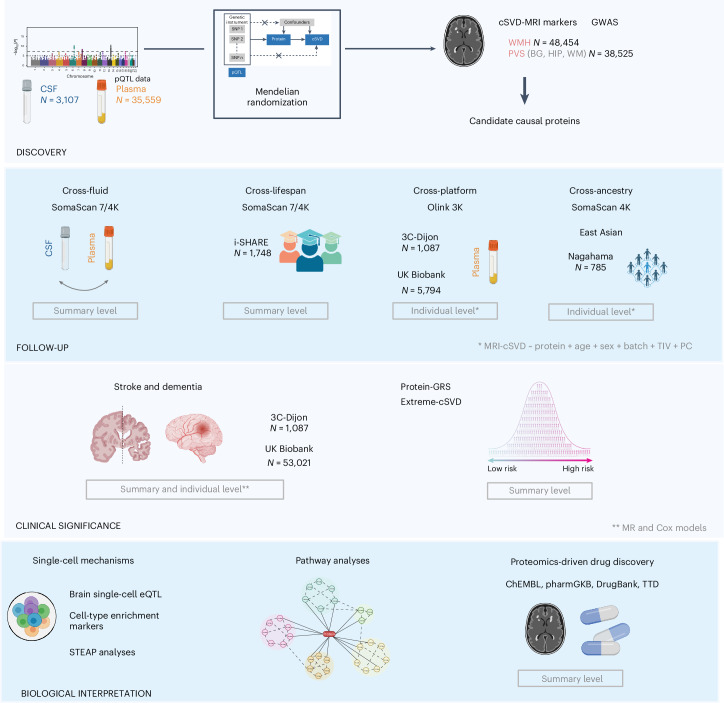
Summary of the analysis plan. ‘Summary level’ and ‘individual level’ correspond to analyses conducted using summary-level-based and individual-level-based datasets, respectively. Created with BioRender.com.

The study design is summarized in Fig. 1.

### Discovery of protein–cSVD associations

We tested genetic associations of CSF and plasma protein levels with MRI-cSVD using 2SMR. We leveraged summary statistics for protein quantitative trait loci (pQTLs) in European-ancestry participants from CSF^12^ (*N* = 3,107; aptamer-based SomaScan 7K assay) and plasma^19^ (*N* = 35,559; SomaScan 5K). *Cis*-genetic instruments were derived for 1,121 CSF and 1,731 plasma proteins, with 721 overlapping proteins (Methods and Supplementary Table 1). For MRI-cSVD, we used the largest published genome-wide association study (GWAS) in European-ancestry participants for WMH volume (*N* = 48,454, mean age 66.0 years)^20^ and PVS burden (*N* = 38,903, 66.3 years)^5^. PVSs were studied separately in three sublocations, white matter (WM), basal ganglia (BG) and hippocampus (HIP), for which association profiles with risk factors and clinical outcomes were previously shown to differ^5^; and which are associated with other MRI-cSVD-related and cSVD-related clinical outcomes^21–27^.

**Fig. 2:**
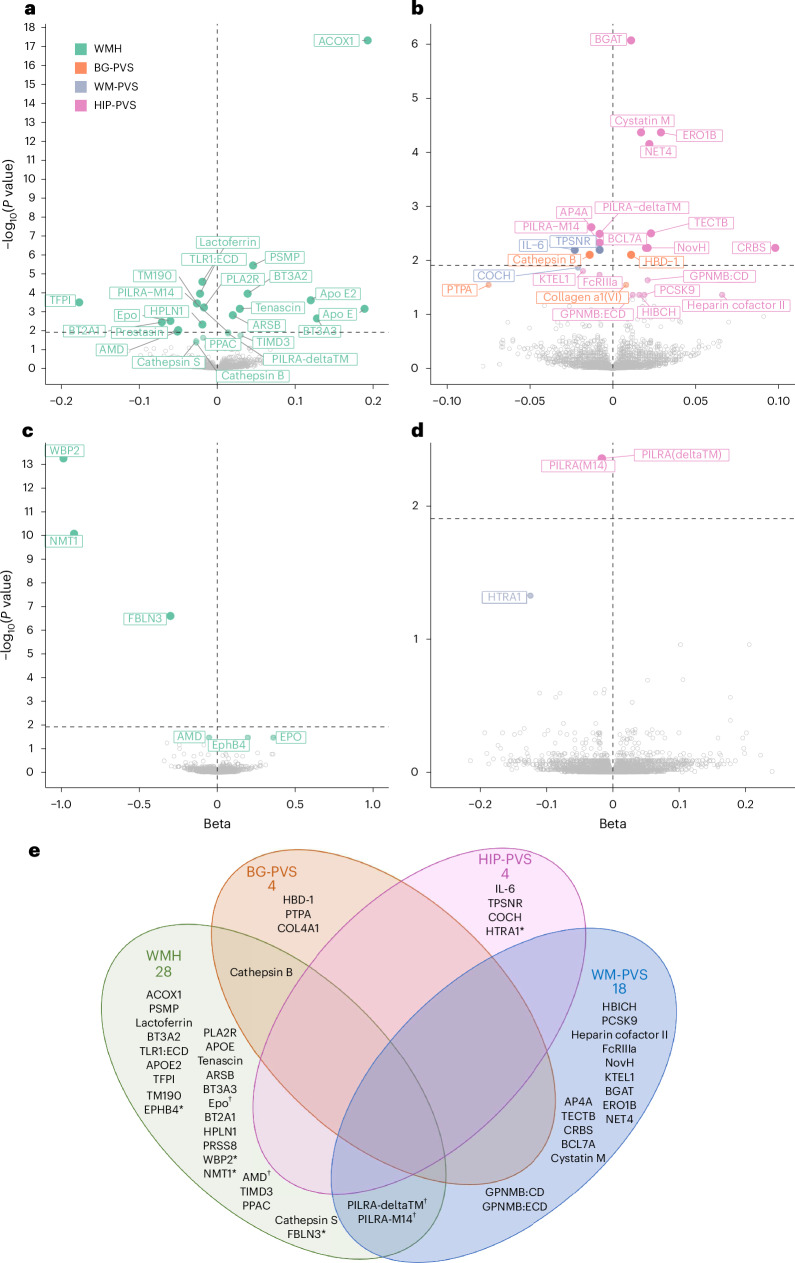
Discovery protein–cSVD associations in CSF and plasma using *cis*-pQTL MR. **a**, Volcano plots of proteins associated with WMHs using *cis*-pQTL MR in CSF. **b**, Volcano plots of proteins associated with PVS burden using *cis*-pQTL MR in CSF. **c**, Volcano plots of proteins associated with WMHs using *cis*-pQTL MR in plasma. **d**, Volcano plots of proteins associated with PVSs using *cis*-pQTL MR in plasma. Each dot represents the MR results for proteins using inverse-variance weighted (IVW) analysis when multiple instrumental variables available, or the Wald ratio when only one instrument was available. Benjamini–Hochberg *P*_FDR_ values are represented in this graph. Represented proteins are significantly associated with MRI marker at *P*_FDR_ (Benjamini–Hochberg FDR threshold) < 0.05. The dashed line in each volcano plot represents the corrected threshold after additionally correcting for the number of phenotypes tested (*P* < 0.0125). **e**, Venn diagram of identified causal proteins associated with MRI-cSVD. An asterisk denotes proteins identified in plasma; a dagger symbol denotes proteins associated in both plasma and CSF; other proteins are associated in CSF.

**Extended Data Fig. 1:**
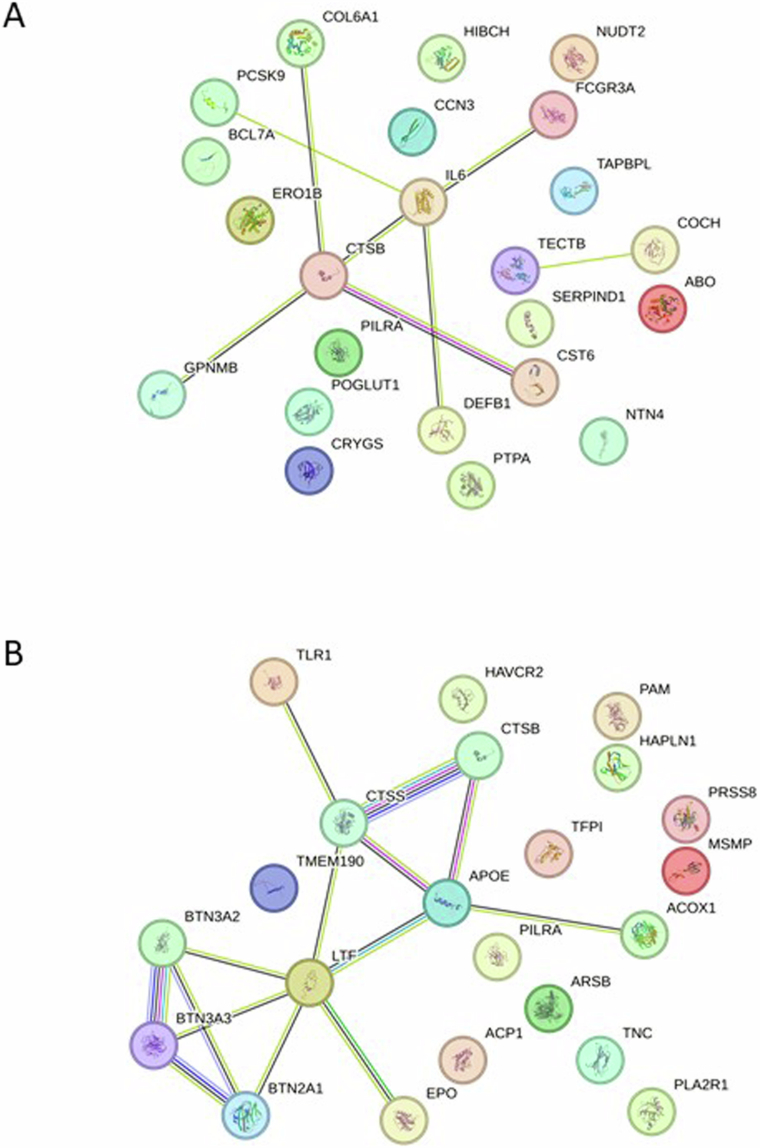
Discovery protein-cSVD associations in CSF and plasma using cis-pQTL mendelian randomization. **A**. String plot of proteins associated with WMH. **B**. String plot of proteins associated with PVS (WM, BG and HIP). Network nodes represent proteins: colored nodes query proteins and first shell of interactors. Edges represent protein-protein associations. Cyan and pink edges are known interactions, cyan: from curated databases, and pink: experimentally determined. Green and blue edges correspond to predicted interactions. Green: gene neighborhood, and blue: gene co-occurrence. Purple corresponds to protein homology, yellow to text mining and black to co-expression.

We identified 46 CSF proteins associated with at least one MRI-cSVD (false discovery rate (FDR)-corrected *P* value (*P*_FDR_) < 0.05): 24 with WMHs, and 25 with PVSs, predominantly in WM (Fig. 2, Extended Data Fig. 1 and Supplementary Tables 2 and 3). Nine plasma proteins were associated with MRI-cSVD (*P*_FDR _< 0.05; WMH: 6, WM-PVS: 2, HIP-PVS: 1) of which four were also significantly associated in CSF (AMD, erythropoietin (EPO; WMH), paired immunoglobulin-like type 2 receptor alpha (PILRA)-M14 and PILRA-deltaTM (WM-PVS); Fig. 2c,d and Supplementary Tables 4 and 5). In both tissues, associations remained robust after sensitivity analyses, except for ACOX1 and WBP2, which showed no evidence of single-variant and fine-mapping-based colocalization and were excluded from further analyses. For three proteins (TFPI, NMT1 and FBLN3), standard single-variant colocalization analyses indicated evidence of colocalization (PP4 ≥ 0.5). However, the fine-mapping-based colocalization using the sum of single effects (SuSiE) yielded more moderate probabilities for TFPI (PP4 = 0.15) and NMT1 (PP4 = 0.48), and a null probability for FBLN3 (PP4 ≈ 0), suggesting a lack of strong support for colocalization in the latter case, warranting cautious interpretation of association results for these proteins (Supplementary Tables 3 and 5). In total, 49 proteins were associated robustly with MRI-cSVD, in CSF (41), plasma (4) or both (4). Three of these were associated with both WMHs and PVSs: cathepsin B (CTSB) and two soluble isoforms of PILRA—deltaTM and M14. Extending these analyses to cerebral microbleeds, we found that higher genetically determined CSF levels of APOE and APOE2 and lower plasma levels of APOE were associated with increased risk of microbleeds (*P*_FDR _< 0.05). CSF and plasma levels of AMD and CSF levels of cystatin M showed nominally significant associations (*P* < 0.05) with microbleeds in plasma (Supplementary Table 6), in the same direction as significant associations with WMHs and WM-PVSs, respectively.

**Extended Data Fig. 2:**
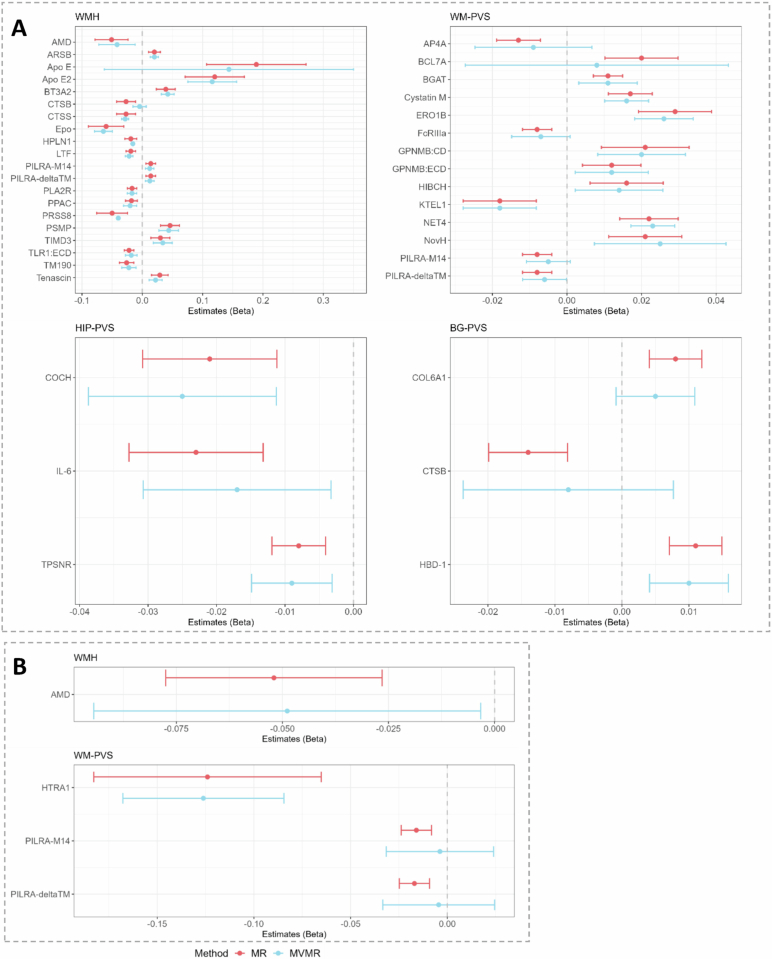
Multivariable Mendelian randomization (MVMR) exploring the modifying effect of systolic blood pressure on the association of A. CSF proteins and B plasma proteins with MRI-cSVD. Only proteins with >1 pQTL available for analysis are represented. Dots correspond to effect estimates (beta) and error bars to 95% confidence intervals. Forest plots correspond respectively to protein associations with WMH, WM-PVS, HIP-PVS and BG-PVS. Red lines correspond to primary MR results; blue lines correspond to multivariable MR (MVMR).

None of the single-variant pQTLs were nonsynonymous variants, which could have resulted in structural changes at the aptamer protein binding site and biased its measurement (Supplementary Table 7). Bidirectional MR ruled out reverse causation, except for an association of larger WM-PVS burden with higher PCSK9 CSF levels (*P*_FDR _= 0.011; Supplementary Table 3). Given the known impact of hypertension on cSVD risk, we conducted sensitivity analyses using multivariable Mendelian randomization (MVMR) accounting for systolic blood pressure (SBP; Methods). Associations with MRI-cSVD were mostly unchanged, but appeared weakened and no longer significant for seven proteins (APOE, CTSB (WMH), AP4A, BCL7A (WM-PVS), COL6A1, CTSB (BG-PVS) in CSF and PILRA-M14 (WM-PVS) in plasma), while maintaining consistent effect directions (Extended Data Fig. 2 and Supplementary Table 8).

The vast majority of protein–cSVD associations revealed previously unreported pathways. A few relate to previous cSVD GWAS findings. Two *cis-*pQTLs were associated with WMH volume at genome-wide significance^20^: for FBLN3 (encoded by *EFEMP1*, chr2p16.1) and NMT1 (chr17q21.31), lower genetically determined plasma levels were associated with larger WMH volume. High-temperature requirement A serine peptidase 1 (HTRA1), of which genetically predicted lower plasma levels were associated with extensive HIP-PVSs, is encoded by a gene harboring both rare mutations causing monogenic cSVD^28^ and common variants associated with ischemic stroke and WMHs^20,29,30^.

**Extended Data Fig. 3:**
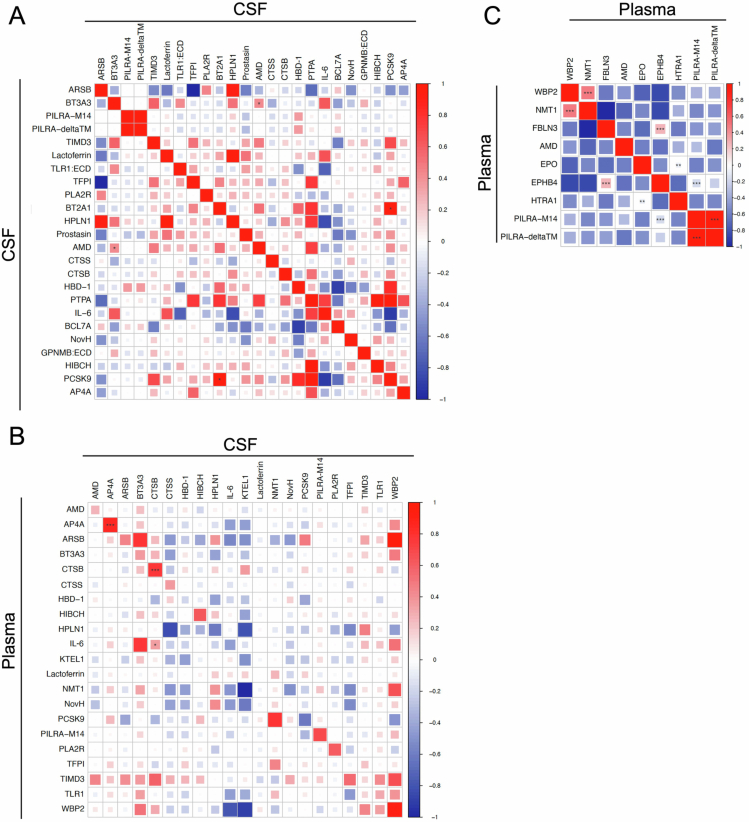
Genetic correlation between proteins associated with MRI-cSVD using protein quantitative trait loci. **A**. Genetic correlation across pQTLs for 24 CSF protein levels associated with MRI-cSVD. **B**. Genetic correlation across pQTLs for 21 CSF or plasma proteins significantly associated with MRI-cSVD and available in both plasma and CSF (x axis: pQTL for CSF protein levels; y axis: pQTL for plasma protein levels). **C**. Genetic correlation across pQTLs for 9 plasma protein levels associated with MRI-cSVD. Genetic correlations were estimated using LD score regression. P-values are based on two-sided Wald tests of the null hypothesis, without adjustment for multiple comparisons. * p < 0.05, ** p < 0.01, *** p < 0.001. Only proteins that converged for genetic correlation analyses are displayed.

Genetically determined levels of cSVD-associated proteins were mostly independent of each other using linkage disequilibrium score regression^31^, except EPHB4 with PILRA-M14 in plasma (genetic correlation (*r*_g _= 0.296; *P* < 5 × 10^−5^; Methods and Extended Data Fig. 3).

### Follow-up and extensions of significant protein–cSVD associations in complementary 2SMR and individual-level data settings

**Fig. 3:**
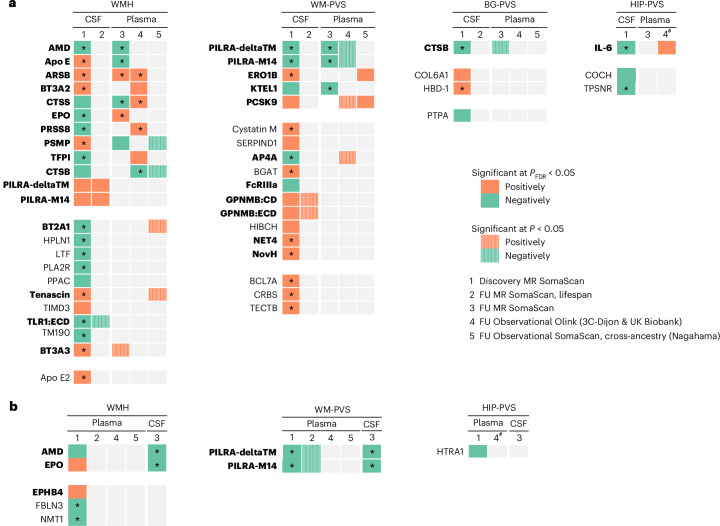
Summary of protein–cSVD associations in discovery and follow-up (lifespan, cross-fluid, cross-platform and cross-ancestry) analyses. **a**, Heatmap of protein–cSVD associations using CSF-based MR analyses as discovery. **b**, Heatmap of protein–cSVD associations using plasma-based MR analyses as discovery. 1. Discovery MR using *cis*-pQTLs from CSF (**a**) and plasma (**b**) and testing their association with WMH volume and PVS burden in the largest meta-analysis of GWAS. 2. Lifespan follow-up MR using *cis*-pQTLs from CSF (**a**) and plasma (**b**) and testing their association with WMHs and PVSs in young adults (i-Share study). 3. Cross-fluid follow-up MR using *cis*-pQTLs from plasma (**a**) and CSF (**b**) and testing their association with WMHs and PVSs in the other fluid than the discovery findings. 4. Cross-platform follow-up using plasma individual-level proteomic data measured with the Olink platform in independent European-ancestry samples (3C-Dijon and UK Biobank studies). 5. Cross-ancestry follow-up using plasma individual-level proteomic data measured with the SomaScan platform in an independent East Asian-ancestry sample (Nagahama study). Columns 1, 2 and 3 represent the direction of effect size from IVW or Wald ratio MR depending on the number of instrumental variables. Columns 4 and 5 represent the direction of effect size from two-sided linear regression analyses adjusted for age, sex, batch, total intracranial volume for WMHs and the first four principal components of population stratification. Dark squares correspond to significant results after FDR correction (*P*_FDR _< 0.05). The asterisk corresponds to significant associations after additional correction for the four phenotypes tested (*P_FDR _< 0.0125). Hatched squares correspond to nominal associations (*P* < 0.05). Orange squares correspond to a positive association (higher protein levels being associated with higher cSVD burden) and green squares correspond to a negative association. FU, follow-up. The hash symbol denotes results of the 3C-Dijon analysis only. Proteins in bold are those showing at least one nominal association (*P* < 0.05) in the same direction in follow-up analyses. The exact *P* values are reported in Supplementary Tables 2 and 9 (**a**) and 4 and 10 (**b**).

We implemented a multipronged follow-up approach across fluids, platforms, ancestries and lifespan (Figs. 1 and 3). For follow-up analyses, significant associations were defined as *P*_FDR_ < 0.05. For cross-ancestry and lifespan approaches, a nominal threshold of *P* < 0.05 was used due to smaller sample sizes and reduced statistical power. First, using 2SMR leveraging summary-based data, we tested whether cSVD–protein associations in CSF showed notable association in plasma, and vice versa, with less stringent multiple-testing correction than in discovery analyses, considering significant associations in the original fluid only. Thirty-seven cSVD-associated CSF proteins had plasma pQTLs available. Nine (24%) were associated with the same MRI-cSVD in plasma (*P*_FDR _< 0.05): APOE, arylsulfatase B (ARSB), EPO, AMD, CTSS, PSMP (WMHs) and PILRA-M14, PILRA-deltaTM, KTEL1 (WM-PVS; Figs. 3a and 6 and Supplementary Table 9). Six cSVD-associated plasma proteins had CSF *cis*-pQTLs available. Four (67%) were associated with the same MRI-cSVD in CSF (*P*_FDR _< 0.05): AMD and EPO (WMHs), and PILRA-M14 and PILRA-deltaTM (WM-PVS; Figs. 3b and 6 and Supplementary Table 10). Directions of association were mostly concordant except for EPO, APOE and PSMP.

**Extended Data Fig. 4:**
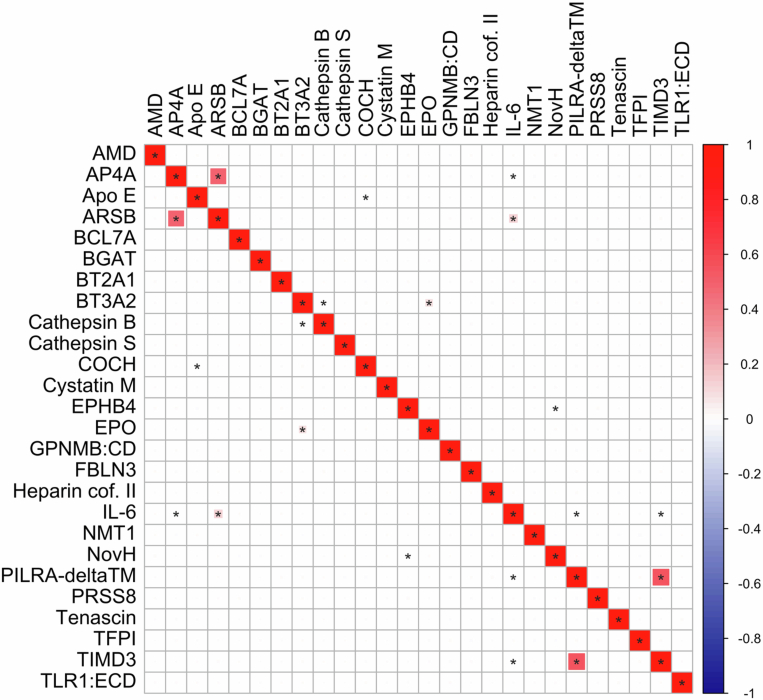
Correlation of protein levels measured in the UKB across the 26 cSVD-associated proteins. Correlations were estimated using Spearman correlation (two-sided). Unadjusted pvalues are displayed. *p < 7.7×10-3 (Bonferroni corrected threshold).

**Extended Data Fig. 5:**
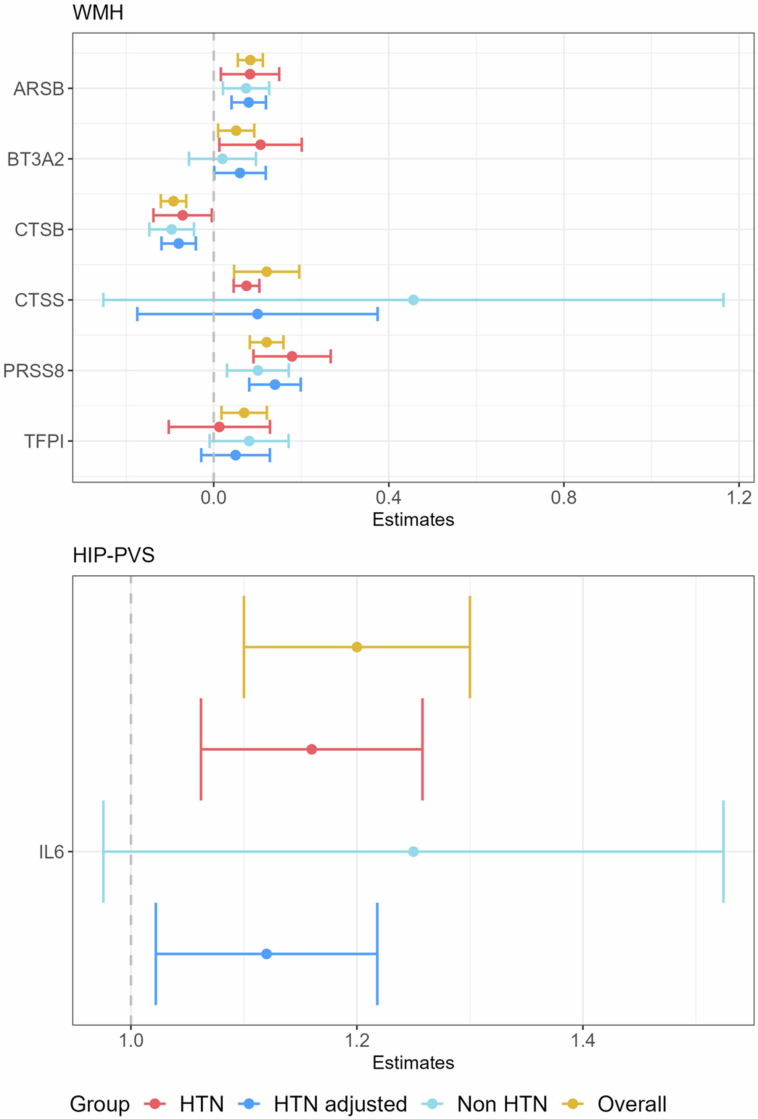
Association of plasma protein levels with MRI-cSVD stratified on hypertension status. Forest plots correspond to protein associations with WMH and HIP -PVS. Results corresponds to meta-analyses of association results in 3C-Dijon and UK Biobank (N = 6,581; 2,088 hypertensive/3,406 non-hypertensive) for WMH; and 3C-Dijon only for HIP-PVS (N = 1,087; 235 hypertensive/852 non-hypertensive), stratified on hypertension status: hypertensive (HTN), non-hypertensive (Non-HTN), all combined (Overall) and adjusted on systolic blood pressure (HTN adjusted). Dots represent effect estimates (beta for WMH and odds ratio for HIP-PVS) and error bars correspond to 95% confidence intervals.

Second, we performed a cross-platform follow-up, testing associations of plasma protein measurements (Olink Explore 3072) with WMH and PVS burden in two independent population-based studies, 3C-Dijon (*N* = 1,087; mean age 72.5 years) and the UK Biobank (*N* = 5,494; 63.5 years), using linear regression of individual-level data (Supplementary Table 11). Twenty-six of 49 cSVD-associated proteins were available and included in meta-analyses of both cohorts (*N* = 6,881; Methods). Seven proteins (27%, all identified in CSF 2SMR) showed associations with the same MRI-cSVD at *P*_FDR_ < 0.05 (ARSB, serine protease 8 (PRSS8), CTSS, CTSB, TFPI and BT3A2 (WMH), IL-6 (HIP-PVS); Figs. 3 and 6 and Supplementary Tables 12 and 13). Directionality was consistent in CSF and plasma for ARSB, CTSB and BT3A2, but not for PRSS8, TFPI, IL-6 and CTSS, all four presenting low correlation (*r* < 0.025, *P* > 0.05) and nonsignificant negative genetic correlation (for IL-6 and TFPI) between tissues (Extended Data Fig. 4 and Supplementary Tables 14 and 15). Inter-platform correlations between SomaScan and Olink were moderate to good in plasma and CSF, respectively (Methods and Supplementary Table 16)^32^. For proteins showing associations with MRI-cSVD at *P*_FDR_ < 0.05 using direct Olink measurements, we ran secondary analyses adjusting for SBP and stratifying on hypertension status (Methods), a key risk factor for MRI-cSVD, thus complementing the aforementioned MVMR analyses. Most associations remained nominally significant after SBP adjustment except for CTSS and TFPI with WMHs. No significant interaction with hypertension status was observed (Supplementary Table 17 and Extended Data Fig. 5).

Third, we conducted a cross-ancestry exploratory follow-up, testing associations of SomaScan plasma protein measurements with WMHs and PVSs using individual-level data from the Japanese population-based Nagahama study (*N* = 785; mean age 68 years). Thirty-eight of the 49 cSVD-associated proteins were available for analyses. Two proteins (identified in CSF in Europeans) were associated with the same MRI-cSVD with consistent directionality at *P*_FDR_ < 0.05 (ERO1B and PCSK9 (WM-PVS)). Nominal associations were observed for four additional proteins (BT2A1, CTSB, TNC and PSMP (WMH); Figs. 3 and 6 and Supplementary Table 18).

Fourth, we took an exploratory lifespan approach by testing the relation of cSVD-associated proteins measured in older adults with MRI-cSVD in young adults (Internet-based Students HeAlth Research Enterprise (i-Share) study, *N* = 1,748; mean age 22.1 years). Using the same *cis*-pQTLs as in the discovery phase, we investigated how genetically regulated protein levels might influence early phenotypic manifestations of cSVD. Consistent with findings in older adults, higher genetically determined CSF levels of PILRA-M14 and PILRA-deltaTM were associated with larger WMH volume at *P*_FDR_ < 0.05. In addition, higher CSF GPNMB:CD and GPNMB:ECD levels were associated with extensive BG-PVS and higher Toll-like receptor 1 (TLR1):ECD CSF levels with larger WMH volume, in a direction consistent with older adults (Fig. 3 and Supplementary Table 19).

Overall, of 49 cSVD-associated proteins identified using 2SMR, (i) 16 CSF proteins showed associations with the same MRI-cSVD in plasma in at least one follow-up analysis at *P*_FDR_ < 0.05, with consistent directionality across fluids for 10, of which 3 also showed lifespan effects (CTSB, PILRA-deltaTM, PILRA-M14); (ii) 24 CSF proteins were not associated with the same MRI-cSVD marker in plasma (*P* ≥ 0.05) and may be considered CSF specific, with 3 of these showing lifespan effects (GPNMB:CD, GPNMB:ECD, TLR1:ECD); (iii) 3 plasma proteins were not associated with the same MRI marker in CSF, and may be considered plasma specific; and (iv) 5 CSF proteins and 1 plasma protein had no follow-up data available^30,33,34^ (Supplementary Table 20 and Figs. 3 and 6).

### Relation of cSVD-associated proteins with stroke and dementia

To assess the clinical implications of the 49 cSVD-associated proteins, we explored their relation with stroke and dementia, using 2SMR and observational survival analyses with individual-level plasma protein measurements (Methods).

**Fig. 4:**
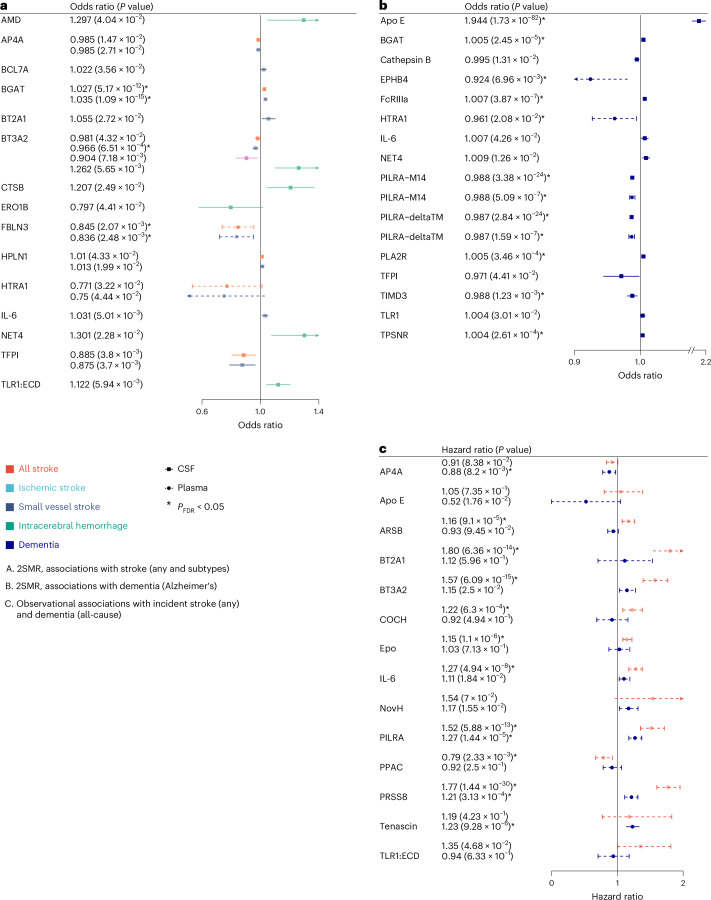
Clinical significance of protein–cSVD findings in CSF and plasma. **a**, Forest plot of protein–cSVD associations with stroke (*N* = 73,652/1,234,808) and its subtypes (ischemic stroke, *N* = 62,100/1,234,808; small vessel stroke, *N* = 6,811/1,234,808; and intracerebral hemorrhage, *N* = 1,545/1,481) using IVW or Wald ratio MR. **b**, Forest plot of protein–cSVD association with Alzheimer’s disease (*N* = 71,880/383,378) using IVW or Wald ratio MR. **c**, Forest plot of protein–cSVD association with stroke and dementia using IVW meta-analysis of two-sided cause-specific Cox models adjusting for age, sex, self-reported ancestry and educational attainment (for incident dementia) of 3C-Dijon and UK Biobank studies (*N* = 54,108; 1,440/1,555 incident stroke and dementia cases). All proteins associated with MRI-cSVD identified in the discovery analysis in CSF and plasma were used for this analysis. Full lines represent proteins measured in CSF. Dashed lines represent proteins measured in plasma. Proteins associated at least at *P* < 0.05 for at least one of the outcomes tested are represented (for stroke, associations with all (sub)types are represented when one or more was significant). Asterisks denote results that are significant after multiple-testing correction (*P*_FDR _< 0.05). Dots correspond to effect estimates (odds ratio (**a** and **b**) and hazard ratio (**c**)), and errors bars correspond to 95% confidence intervals.

For 2SMR, we leveraged the aforementioned CSF and plasma pQTLs and European-ancestry summary statistics of GWAS for stroke and its subtypes (*N* ≤ 73,652 cases) and dementia (*N* = 71,880 cases; Methods). Twenty-four proteins (49%) showed associations with at least one clinical outcome at *P* < 0.05 (Figs. 4 and 6). At *P*_FDR_ < 0.05, eight CSF proteins (APOE, PILRA-M14, PILRA-deltaTM, FcRIIIa, BGAT, PLA2R, TIMD3, TPSNR) and four plasma proteins (EPHB4, HTRA1, PILRA-M14, PILRA-deltaTM) were associated with dementia, while one CSF protein (BGAT) and one plasma protein (FBLN3) were associated with any stroke and ischemic stroke (Supplementary Tables 20–22).

For observational survival analyses, 1,087 and 53,021 participants with 40/84 and 1,400/1,471 incident stroke (any)/dementia (all-cause) cases were available in the 3C-Dijon and UK Biobank population-based cohorts, with measurements for 24 of 49 cSVD-associated proteins. Association statistics from Cox cause-specific models were meta-analyzed (Methods; *N* = 54,108; 1,440/1,555 incident stroke and dementia cases). Fourteen proteins showed an association with risk of stroke or dementia at *P* < 0.05, of which 11 were at *P*_FDR_ < 0.05. Four of the latter also reached *P* < 0.05 in 2SMR analyses (PILRA, for stroke and dementia, and BT2A1, BT3A2 and IL-6 for stroke), while 7 were significant only in observational analyses (5 for stroke: ARSB, COCH, EPO, PPAC and PRSS8; 3 for dementia: AP4A, PRSS8 and tenascin; Fig. 4c and Supplementary Tables 20 and 23).

**Extended Data Fig. 6:**
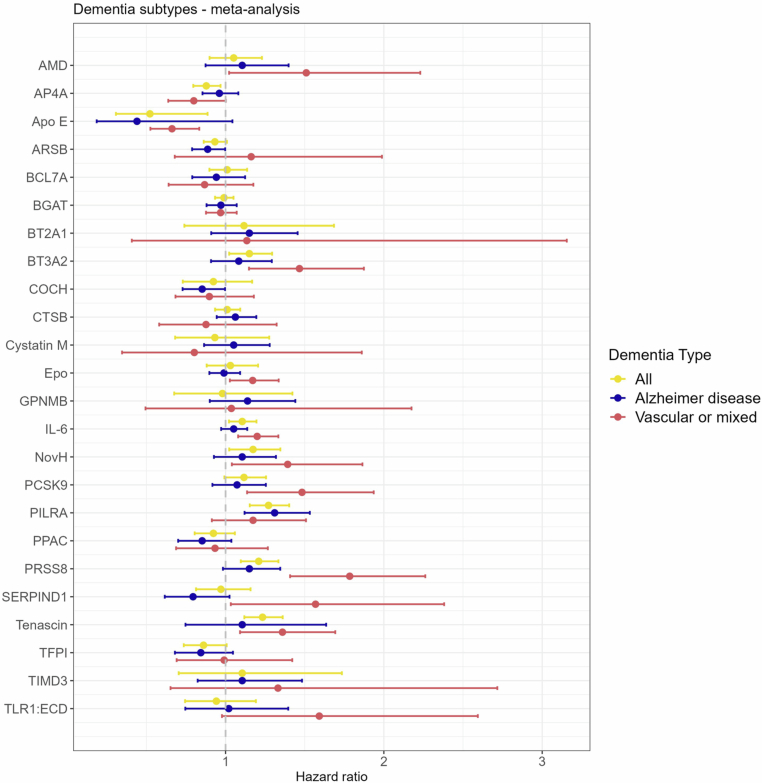
Protein-cSVD associations with dementia subtypes (vascular and Alzheimer’s disease): meta-analysis of 3C-Dijon and UK Biobank. Results of cause-specific Cox models (Methods). N-vascular dementia=385; N-Alzheimer=1,107). Dots represent hazard ratios and errors bars corresponds to 95% confidence intervals.

Overall, 30 cSVD-associated proteins (61%) were associated at *P* < 0.05 and 18 at *P*_FDR_ < 0.05 with stroke or dementia (both for PILRA and PRSS8). Fine–Gray sensitivity analyses were consistent with the main findings, except for APOE, of which the observed effect was attenuated and no longer significant (*P* > 0.05; Supplementary Table 24). All proteins associated with dementia were significant only for vascular or mixed dementia subtypes in secondary analyses (Methods), except for PILRA, whose association with dementia appeared to be driven by Alzheimer’s disease (Supplementary Table 25 and Extended Data Fig. 6).

**Extended Data Fig. 7:**
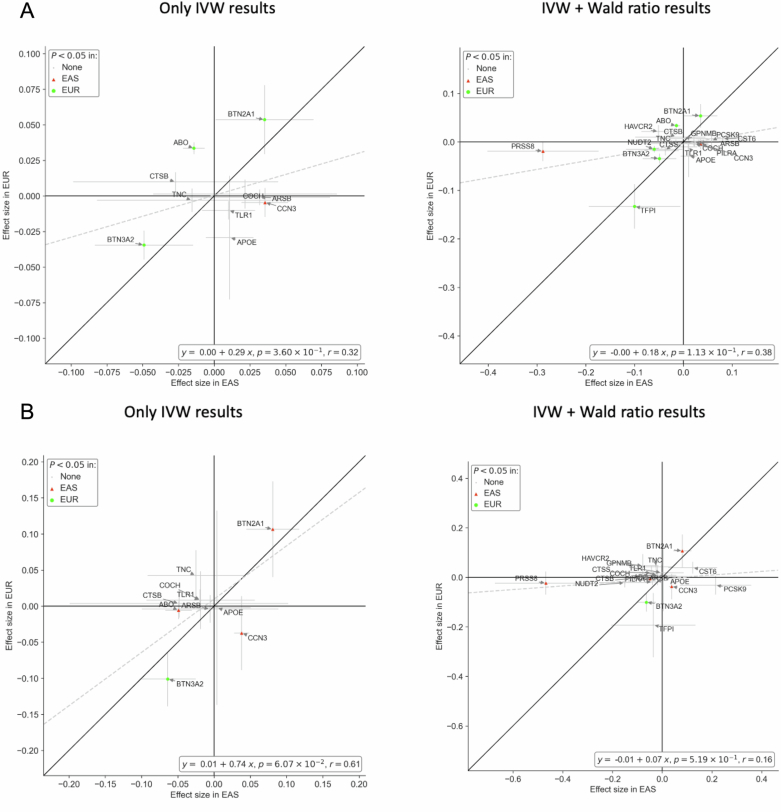
Comparison of effect size estimates for MR associations of cSVD-associated proteins with ischemic stroke and small vessel stroke between Europeans (EUR) and East-Asians (EAS). **A**. Ischemic Stroke. **B**. Small vessel Stroke.

Nineteen of 49 cSVD-associated proteins were available for follow-up in East Asian participants in relation with stroke using 2SMR, leveraging plasma pQTLs from BioBank Japan (BBJ; *N* = 2,886) and an East Asian stroke GWAS (*N* ≤ 17,493). Despite a substantially smaller sample size for exposure and outcome than in the European ancestry, correlation of effect sizes was moderate to high (Extended Data Fig. 7). Higher plasma levels of NovH, an extracellular matrix (ECM)-associated protein involved in cardiovascular development, were associated with increased risk of small vessel stroke at *P*_FDR_ < 0.05 (Supplementary Table 26).

### Biological interpretation

In pathway enrichment analyses using FUMA, cSVD-associated proteins were enriched in proteoglycan binding and ECM proteins (*P*_FDR _< 0.05; Supplementary Table 27a). Among cSVD-associated proteins in CSF, proteins involved in immune response activation and signaling regulation were overrepresented (*P*_FDR _< 0.05; Supplementary Table 27a).

**Extended Data Fig. 8:**
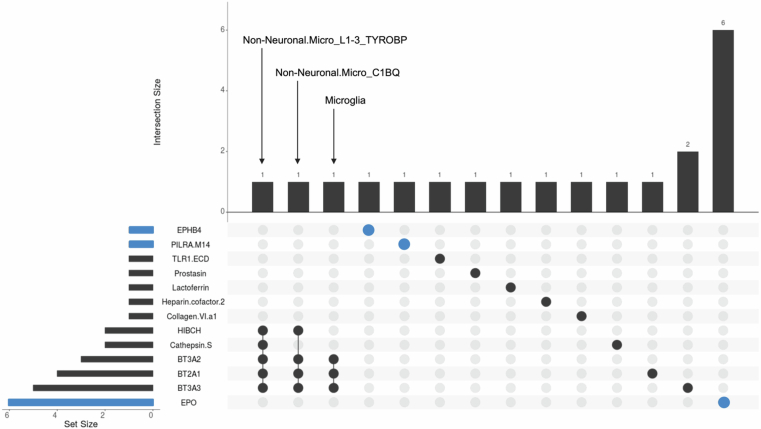
Cell-type enrichment in single cell RNA-seq databases using STEAP. Upset plot displays the number of significant enrichment results by protein (pQTL) horizontally and by cell-type vertically. CSF pQTLs are in black and plasma pQTLs are in blue. Details are displayed in Supplementary Table 25. Human and mouse single-cell databases are used in this analysis (Methods).

**Extended Data Fig. 9:**
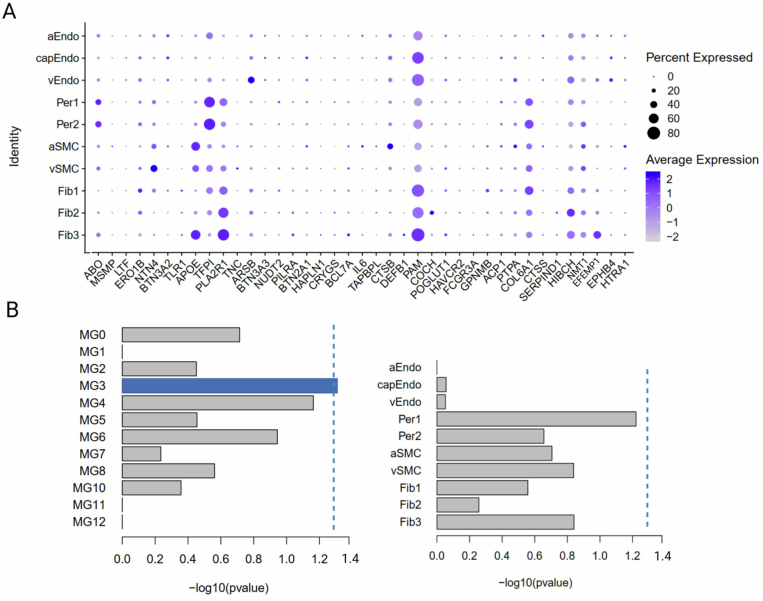
Single-nucleus gene expression/enrichment analyses. **A**. Single-nucleus cerebrovascular gene expression data of cSVD-associated protein coding genes in dorsolateral prefrontal cortex (ROS-MAP study). **B**. Enrichment analyses of cSVD-associated protein coding genes in microglial states and vascular cells using in silico vascular enrichment (two-sided). Unadjusted pvalues are displayed. Dotted line corresponds to pval<0.05.

To explore cell specificity of protein–cSVD associations, we conducted single-cell enrichment analyses using the single-cell-type enrichment analysis for phenotypes (STEAP) tool, leveraging publicly available single-cell sequencing resources (Methods and Supplementary Table 28). Genes encoding cSVD-associated proteins were significantly enriched in microglia for several CSF proteins (BT2A1, BT3A2, BT3A3, CTSS, HIBCH) and in immune cells for plasma protein EPO (Supplementary Table 29 and Extended Data Fig. 8). Next, we used single-nucleus RNA sequencing (RNA-seq) in up to 443 postmortem brain samples (dorsolateral prefrontal cortex) from the ROSMAP population-based cohort^26–29^. Cell-type-specific brain expression quantitative trait loci (eQTLs) could be derived for 19 and 10 genes encoding cSVD-associated proteins in nonvascular and vascular brain cells, respectively (Methods and Supplementary Tables 30 and 31). Using 2SMR, three associations were observed (*P*_FDR _< 0.05) with evidence for colocalization: lower expression of *TLR1* in oligodendrocytes and *CTSS* in smooth muscle cells was associated with larger WMH volume (same direction as in CSF); higher expression of *ABO*, encoding BGAT, in pericytes was protective for extensive WM-PVSs (opposite direction compared to CSF). Genes encoding cSVD-associated proteins showed a nonsignificant trend toward enrichment in pericytes and significant enrichment in a microglial state type overrepresented in processes such as ribosome biogenesis, amyloid fibril formation and regulation of T cell-mediated immunity (Extended Data Fig. 9)^35^.

### Proteomics-driven drug discovery

**Fig. 5:**
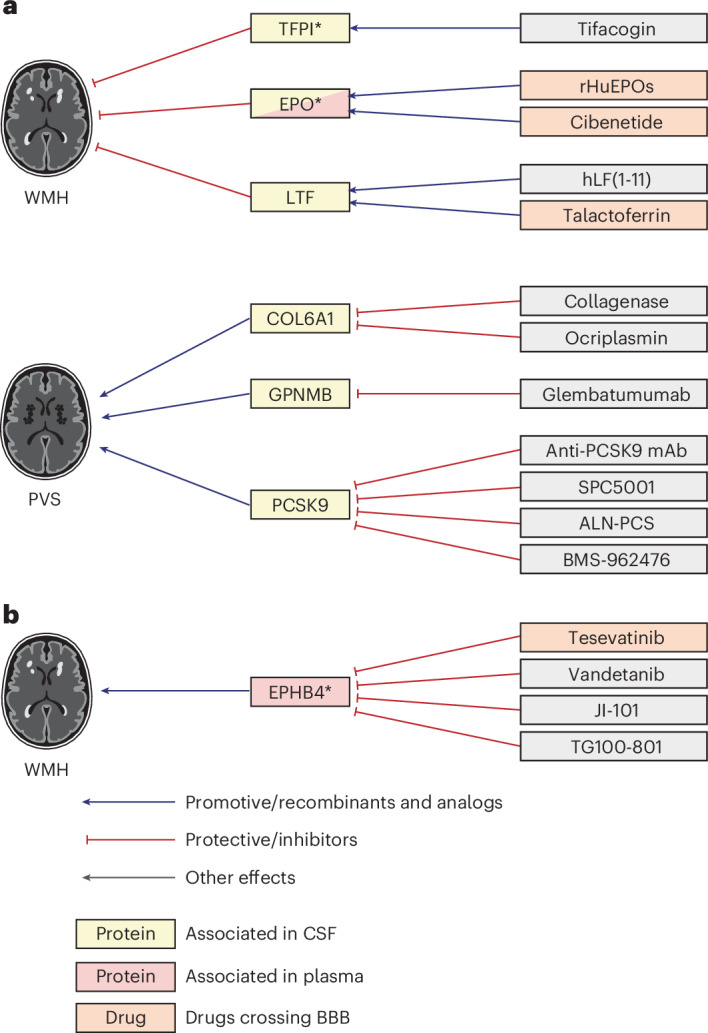
Proteomics-driven drug discovery. **a**, Drug-discovery analysis conducted using CSF protein–cSVD MR IVW or Wald ratio estimates for WMH and PVS findings. **b**, Drug-discovery analysis conducted using plasma protein–cSVD MR IVW or Wald ratio estimates for WMHs. Proteins in yellow correspond to proteins associated with the MRI-cSVD marker in CSF and in red in plasma, in discovery analyses. An asterisk denotes proteins with associations in at least one of the follow-up modalities (at *P* < 0.05). Red arrows correspond to a protective effect of a protein on MRI-cSVD (reducing cSVD burden) or an inhibitory effect of a drug on the cSVD-associated protein; blue arrows correspond to a deleterious effect of a protein on MRI-cSVD (promoting cSVD burden) or an analog effect of a drug on the cSVD-associated protein. Drugs in orange cross the BBB. mAb, monoclonal antibody.

**Fig. 6:**
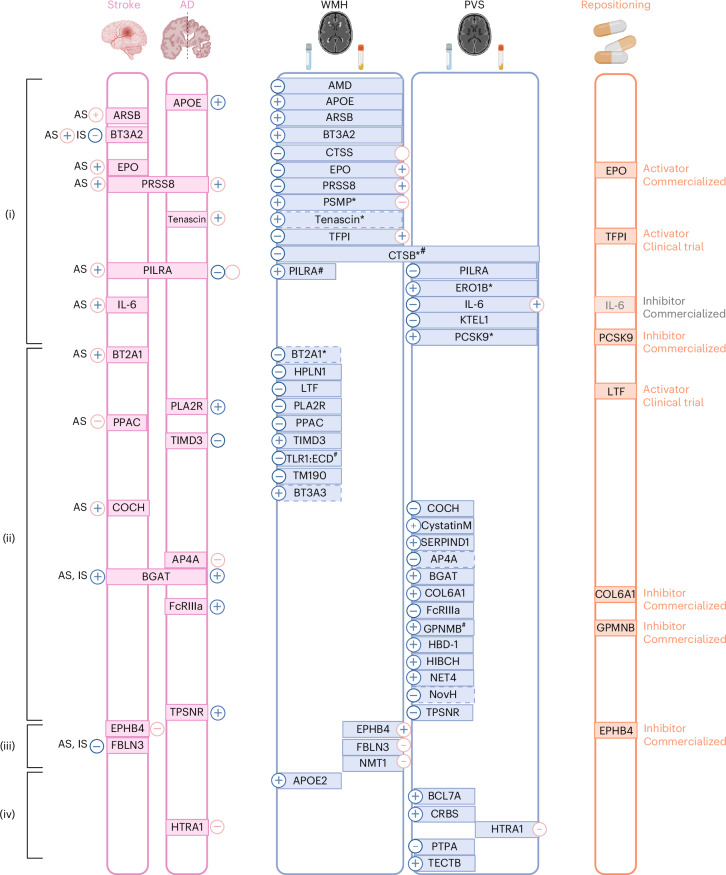
Integrated summary of our findings. Proteins associations with WMH, PVS or both are represented in the middle. For each MRI marker, the left side corresponds to CSF findings and the right side to plasma findings. An asterisk denotes proteins with cross-ancestry association. A hash symbol denotes proteins with lifespan association. Associations with stroke, dementia or both (*P*_FDR _< 0.05) in either MR or observational analysis are represented on the left of the figure. Subtypes of stroke are as follows: AS, any stroke; IS, ischemic stroke. Minus and plus signs correspond to the direction of association referring to higher level of the protein. Blue plus or minus signs correspond to findings in CSF and pink in plasma. Empty plus or minus signs correspond to a situation where opposite directions were observed in the same tissue using MR and observational study. Drug repositioning is represented on the right of the figure. (i) Proteins associated with the same MRI-cSVD marker in cross-fluid follow-up (for cSVD-associated proteins identified in CSF discovery: showing significant association in plasma follow-up; for cSVD-associated proteins identified in plasma discovery: showing significant association in CSF follow-up); (ii) CSF-specific proteins (showing no significant association in plasma follow-up); (iii) plasma-specific proteins (showing a nonsignificant association in CSF follow-up); (iv) no follow-up available. AD, Alzheimer’s disease. Created with BioRender.com.

We used MR estimates from the 49 cSVD-associated proteins to support drug discovery. Using public drug databases (Methods), we curated drugs (commercialized for other indications or under investigation in clinical trials) targeting these proteins in a direction compatible with beneficial therapeutic effects against cSVD. We identified such drugs for EPO, lactoferrin (LTF), TFPI and EPHB4 for WMHs; GPNMB and PCSK9 for WM-PVSs and COL6A1 for BG-PVSs (Figs. 5 and 6 and Supplementary Tables 20 and 32). Some of these proteins have predicted or experimentally demonstrated interactions with each other (Fig. 2e), suggesting that identified drugs may impact related pathways. Of note, although this may not necessarily be required to treat cSVD, drugs targeting EPO and LTF as agonists and EPHB4 as inhibitors cross the blood–brain barrier (BBB; Supplementary Table 32). The association of plasma PCSK9 with WM-PVSs was independent of low-density lipoprotein cholesterol and triglyceride levels in UK Biobank (Supplementary Table 12b).

Results of protein–cSVD associations along with clinical significance, enrichment analyses and drug target identification are summarized in Supplementary Table 20 and Fig. 6.

## Discussion

We describe a comprehensive biological fingerprint of cSVD, comprising 49 protein–cSVD associations, predominantly in the CSF, by integrating unique CSF and plasma pQTL resources with the largest GWAS of MRI-cSVD in an MR framework. We implemented a multipronged follow-up approach, across fluids, proteomic platforms and ancestries, using both MR and observational analyses. Sixteen proteins were associated with MRI-cSVD in both CSF and plasma, several of which were both in Europeans and East Asians, while 24 and 3 proteins were associated in CSF or plasma only. Pathway and cell-type enrichment analyses suggest an important role of ECM and immune response pathways, with single-cell RNA-seq analyses showing enrichment in microglia and a specific microglial state. Strikingly, several cSVD-associated proteins involved in immune response regulation already showed associations with MRI-cSVD at age 20 with consistent directionality. Over half of cSVD-associated proteins showed at least nominal associations with stroke, dementia or both, highlighting their clinical relevance. Importantly, our findings also provide genetic support for repositioning of drugs targeting seven cSVD-associated proteins in a direction compatible with beneficial therapeutic effects.

While a few proteins are encoded by genes within cSVD GWAS loci (NMT1, FBLN3, HTRA1, APOE, TFPI), shedding new light into underlying biological mechanisms^20,33^, most associations are distinct from those previously reported. IL-6 was previously associated with WMH volume^17^, but its association with HIP-PVS was not shown before. Earlier cSVD proteomic studies focused on limited protein panels^36,37^, mostly in plasma^14–18^ and on smaller cohorts (*N* < 1,000)^38^, with one recent study on 16 CSF proteins^39^. Here, we analyzed >2,000 plasma and CSF proteins with *cis*-pQTLs in >40,000 participants. CSF biomarkers have become crucial for understanding neurodegenerative and neuroinflammatory mechanisms given their proximity to the central nervous system^40–42^, and our findings suggest this also applies to neurovascular diseases like cSVD. CSF-based MR revealed five times more protein–cSVD associations than plasma-based MR, despite a tenfold smaller sample size. Among proteins with pQTLs in both compartments, 67% of plasma cSVD-associated proteins were also associated in CSF, whereas only 24% of CSF-associated proteins showed plasma associations; adding follow-up with individual-level plasma measurements yielded 43% concordance between protein–cSVD associations in CSF and plasma, suggesting some protein–cSVD associations are CSF specific, as seen in other neurological disorders^12,13^.

Some protein–cSVD associations were particularly robust and consistent across fluids, platforms, 2SMR and observational analyses, especially PILRA-deltaTM, PILRA-M14 and CTSB, associated with WMHs and PVSs, and ARSB, associated with WMHs. Notably, the association of one PILRA isoform with WM-PVS and of CTSB with WMHs and BG-PVS lost significance in MVMR adjusting for SBP. CTSB, whose gene is located at a known SBP risk locus^43^, remained associated with WMHs in observational analyses after blood pressure adjustment, suggesting additional mechanisms.

PILRA is a microglial immunoreceptor involved in amyloid-β (Aβ) uptake and herpes simplex virus 1 infection^44^. SomaScan measures soluble PILRA isoforms lacking the transmembrane domain^45^ (PILRA-deltaTM and PILRA-M14), while Olink detects the full protein. Higher CSF levels of PILRA-M14 and PILRA-deltaTM (2SMR, SomaScan) were associated with larger WMH volume, whereas higher CSF and plasma PILRA-M14/deltaTM (2SMR, SomaScan) and plasma PILRA (observational, Olink) were associated with smaller WM-PVS burden, potentially reflecting a protective effect on the cerebral amyloid angiopathy subtype of cSVD, of which WM-PVS is a marker^46^. Consistent with WMH associations, higher plasma PILRA was associated with increased risk of incident stroke and dementia (observational), but in 2SMR higher plasma and CSF PILRA-M14/deltaTM were associated with lower dementia risk. Possible explanations include isoform-specific effects or differences in dementia definitions (2SMR: Alzheimer’s disease; observational: all-cause dementia). Previous work supported PILRA as the likely causal gene at the chr7q21 Alzheimer’s disease risk locus, suggesting a common missense variant (rs1859788, *r*^2^ = 0.3 with PILRA pQTLs) protects against Alzheimer’s disease via reduced inhibitory signaling in microglia and lower herpes simplex virus 1 infection during recurrence^47^.

CTSB is a cerebrovascular matrisome protein identified in brain microvessels^37^. This lysosomal cysteine protease is involved in proteolysis of ECM components and enhanced vessel wall permeability^48^, and in proteolysis of amyloid precursor protein, implicated in Alzheimer’s disease^49^. Using 2SMR, lower CTSB levels in CSF were associated with larger WMHs and BG-PVSs, replicating in plasma and importantly also across platforms (2SMR and observational) and ancestries (Europeans and East Asians). Lower CTSB levels were also significantly associated with higher risk of dementia (2SMR). Mutations in *CTSA* (encoding cathepsin A) cause a rare monogenic autosomal recessive cSVD known as CARASAL^50^. Our findings now expand the involvement of cathepsins to complex cSVD.

ARSB plays an important role in ECM degradation, regulation of neurite outgrowth and neuronal adaptability in the brain^51^, where it is expressed predominantly in microglia^52,53^. While ARSB deficiency causes a lysosomal storage disorder^54^, here higher CSF and plasma ARSB levels were associated with greater WMH volume (2SMR and observational), making ARSB a compelling candidate to explore as a potential biomarker of cSVD. This will require absolute quantification assays and testing of the predictive and prognostic potential of circulating ARSB levels.

Our proteogenomic analyses thus lend support to a prominent role of the matrisome in cSVD, corroborating and expanding findings from large genomic studies^5,6^ and preclinical work on monogenic cSVD^37^, by revealing numerous matrisome proteins not previously implicated. PRSS8, associated with WMH, also shows a highly significant association with risk of incident stroke and dementia in observational analyses. Moreover, while matrisome protein HTRA1 is known to play a central role in cSVD, both monogenic^55^ and multifactorial^30,34^, our results reveal an association of lower HTRA1 plasma levels with extensive HIP-PVS, and, at nominal significance, with dementia. This expands recent descriptions of loss-of-function mechanisms for HTRA1 associations with ischemic stroke and coronary artery disease risk^30,34^.

Our findings reveal associations of immune response pathways with MRI-cSVD. Enrichment in immune regulation was prominent for CSF proteins, and integration with single-cell data highlighted microglial cells, the brain’s primary resident immune cells, particularly a microglial state overrepresented in T cell-mediated immunity and amyloid fibril formation. This provides molecular insights into immune activation in cSVD, complementing smaller biomarker studies and MRI–histopathology data linking WMHs to microglial activation^56–61^. Notably, our results suggest that immune regulation could be one of the earliest processes involved in cSVD, as demonstrated for Alzheimer’s disease^62^. Of all cSVD-associated proteins, PILRA isoforms (microglial immunoreceptor), TLR1 (involved in activation of innate immunity) and GPNMB (transmembrane glycoprotein upregulated upon tissue damage and inflammation) associations were already detectable in young adults in their twenties, in directions consistent with older adults.

This work unveiled emerging prospects for drug repositioning for cSVD, with the identification of multiple drugs targeting seven cSVD-associated proteins (EPO, LTF, TFPI, EPHB4, COL6A1, GPNMB and PCSK9). Based on the protective effect of higher genetically determined CSF EPO levels on WMH volume, potential repositioning of EPO analogs crossing the BBB and studied in phase II clinical trials for other indications (depression, neuropathy) is a compelling example. EPO, besides stimulating erythropoiesis, is a neuroprotective protein acting through the Keap1–Nrf2 pathway to protect from ischemia–reperfusion injury^63^ and by stimulating neurogenesis. Neuroprotective effects are conserved for EPO derivatives lacking erythropoiesis-stimulating activity^64^. In contrast with CSF EPO, high plasma EPO levels were associated with larger WMH volume. This likely reflects distinct sources and regulation of EPO in CSF and plasma. While the major EPO-producing cells in the brain are pericytes^65,66^, most EPO is produced in the kidney. As EPO does not cross the BBB, plasma EPO likely mostly reflects the kidney production. LTF, of which higher genetically determined CSF levels were associated with smaller WMH volume, also has neuroprotective and anti-inflammatory properties^67,68^ and, interestingly, shows strong protein–protein interactions and collaborative anti-inflammatory properties with EPO^63^. LTF agonists are currently tested in phase I and III trials for sepsis and cancer. Optimized versions of EPO and LTF have shown experimental evidence of neuroprotective effects in ischemic stroke and intracerebral hemorrhage^69–71^. Other repositioning candidates for cSVD include drugs inhibiting PCSK9. In addition to significant 2SMR associations between higher CSF PCSK9 levels and larger WM-PVS burden, higher plasma PCSK9 levels were also associated with extensive WM-PVS in observational analyses in the East Asian Nagahama cohort and the UK Biobank, independent of lipid levels in the latter. A protective effect of PCSK9 inhibitors on any ischemic stroke has been demonstrated^72^ but has not been shown specifically for cSVD. Experimental work has shown PCSK9 to regulate Aβ clearance from the brain^73^, and peripheral PCSK9 inhibition to reduce Aβ pathology in the prefrontal cortex and HIP in mice^73^. Intriguingly, bidirectional MR also showed an association of genetically determined larger WM-PVS burden with higher CSF PSCK9 levels. Although speculative, this could potentially reflect an influence of glymphatic dysfunction, of which PVSs are thought to be a marker, on PCSK9 clearance from the brain^74^.

As reported by others^11,12^, for six proteins we observed opposite directionality of associations for plasma versus CSF protein levels (APOE, EPO, PSMP, PRSS8 and TFPI with WMHs, IL-6 with HIP-PVS, and BT3A2 with stroke). As for EPO, differences in protein production and regulation between CSF and blood, or exchanges from CSF to plasma, may explain distinct effects on cSVD^11^. This underscores the value of multi-compartment proteomic analyses and warrants further study of underlying mechanisms.

We acknowledge limitations. Discovery was restricted to proteins quantified by SomaScan, for which *cis*-pQTLs could be derived, representing less than 10% of known proteins. Moreover, we were underpowered to conduct discovery association analyses with cerebral microbleeds, but performed exploratory analyses with WMH- and PVS-associated proteins. pQTLs were derived from a population enriched in neurologically impaired individuals (especially with Alzheimer’s disease); however, we previously showed that pQTLs are only marginally influenced by disease status^12^; moreover, follow-up samples were not enriched in individuals with Alzheimer’s disease. The unique CSF proteomics resource we used was crucial to derive a biological fingerprint of cSVD; however, no additional CSF proteogenomics resource was available for follow-up. Nevertheless, the multipronged follow-up and extension across fluids, platforms, lifespan and ancestries enhances the robustness of our findings and their transportability to East Asian populations where cSVD is particularly prevalent^75^. Four plasma protein–cSVD associations discovered using 2SMR either were not significant in follow-up observational analyses or had no follow-up available. While in some instances this may reflect spurious associations, it could also be explained by lack of power or modest correlation across platforms as previously reported^32,76,77^. In fact, two of these proteins showed significant associations with stroke or dementia (FBLN3, HTRA1) in consistent direction and three were located within cSVD GWAS loci (FBLN3, HTRA1, NMT1), supporting the robustness of these findings. Inconsistent directionality of significant associations between pQTL analyses (SomaScan) and direct measurements (Olink) for two proteins (CTSS with WMH and PILRA with dementia) requires further exploration but could reflect that distinct isoforms are being captured as suggested by others^32,76,77^. Importantly, associations of genetically proxied CTSS and PILRA levels with MRI-cSVD were consistently observed in plasma and CSF, and across the lifespan, in independent datasets, supporting the robustness of these results. We also acknowledge that our lifespan approach is limited by the use of pQTL data derived from older populations due to the current lack of genetic instruments available for younger individuals, although international efforts to address this gap are ongoing. Single-cell analyses were conducted independently on each cell-type and protein, as we are not equipped to assess how these different cell types and proteins may interact with each other. When larger single-cell eQTL datasets become available, future studies should address these questions.

In conclusion, our large-scale proteogenomic study provides a comprehensive in vivo biological fingerprint of cSVD, with 49 protein–cSVD associations, mostly in the CSF. The results highlight important biological processes underlying cSVD at the molecular and cellular levels and point to early life mechanisms involving immunity and inflammation. They also pave the way for deriving circulating biomarkers and drug repositioning for cSVD with concrete development opportunities, an important step forward for a highly prevalent condition with no specific biomarker and treatment to date.

## Methods

This study complies with all relevant ethical regulations, and all participants gave written, informed consent (Supplementary Methods).

### Discovery of protein–cSVD associations

#### Deriving genetic instruments for circulating protein levels (instrumental variables for the exposure) using pQTLs

pQTLs were generated from GWAS of circulating protein levels. CSF pQTL summary statistics were obtained from 7,028 proteins (SomaScan 7K platform; *N* = 3,107, European ancestry); 1,076 participants were cognitively normal, 1,001 had clinically determined late-onset Alzheimer’s disease, 118 had early-onset Alzheimer’s disease, 281 non-Alzheimer’s disease dementia and 631 had Parkinson’s disease^12^. Plasma pQTL summary statistics were obtained from 4,907 proteins (SomaScan 5K platform; *N* = 35,559 European ancestry, cognitively normal) from either the Icelandic cancer project (52%) or deCODE genetics (48%)^19^. *cis*-pQTLs were defined as genetic variants within 1 Mb of the corresponding protein-coding gene. Genetic variants were selected based on genome-wide significant associations (*P* < 5 × 10^−8^) with protein abundance after clumping using PLINK2 (ref. ^78^) for linkage disequilibrium at *r*^2^ < 0.01, within 1 Mb using the European 1000 Genome reference panel. Genetic variants included in the major histocompatibility complex region (chromosome 6: 26–34Mb) were removed considering the complex linkage disequilibrium structure of the region. The strength of the instrumental variables was measured using the *F*-statistic (*F*-statistic > 10 was considered strong). Following these steps, we selected up to 1,121 CSF and 1,732 plasma proteins with *cis*-acting pQTLs for MR analyses.

#### Genetic associations with MRI-cSVD (outcome)

We used summary statistics from the latest GWAS meta-analyses of WMH volume, in 48,454 participants (mean age 66.0 years), and of extensive PVS burden in WM, BG and HIP, in up to 38,903 participants (mean age 68.3 years), from the general population, of European ancestry, and free of stroke^5,20^. In exploratory analyses, we examined the relation of identified WMH- or PVS-associated proteins with cerebral microbleeds leveraging the only available GWAS, with limited statistical power (*N* = 25,862; 3,556 cases)^79^. Cohorts from which the pQTLs were derived were not included in WMH, PVS or microbleed GWAS meta-analyses.

#### Analytical steps for MR analyses

We applied 2SMR analyses using the ‘TwoSampleMR’ package (v.0.5.6)^80^ to assess the causal association between genetically predicted CSF and plasma protein levels and MRI-cSVD. pQTLs obtained after instrument selection for each protein were used as instrumental variables. We extracted the association estimates between the variants and the exposures or the outcomes and aligned the effect alleles. For proteins with multiple instrumental variables, we computed MR estimates with random-effect IVW analysis^81^ relying on distinct assumptions for validity: (i) Heterogeneity across the MR estimates was assessed for each instrument using Cochran’s *Q* statistic (*P* < 0.05 was considered significant)^81^; (ii) Horizontal pleiotropy was assessed using the MR-Egger intercept as a measure of directional pleiotropy (*P* < 0.05 was considered significant)^82^. We further conducted various sensitivity analyses^83^:

1. The identification of outlier instrumental variables and their removal from analyses was conducted using MR pleiotropy residual sum and outlier (MR-PRESSO)^84^ (*P* < 0.05 was considered significant).
2. Reverse MR was run by reversing the direction of inference, using the MRI-cSVD markers as the exposure and proteins as the outcome, to formally rule out reverse causation.
3. MR-Egger regression^85^ and weighted median, which are more robust to the use of pleiotropic instruments, were used. When pleiotropy was observed, we retained results when at least two of the three sensitivity methods (MR-Egger, weighted median, MR-PRESSO) were concordant with each other and *P* < 0.05.
4. MVMR^86,87^ estimating the direct effect of multiple exposures, that is, simultaneously including in the same model genetic instruments for protein levels and for SBP to rule out confounding of protein–cSVD associations in SBP GWAS in European ancestry participants (*N* = 757,601)^43^.

For proteins with a single instrumental variable, we computed MR estimates using the Wald ratio, followed by colocalization analyses using coloc^88^, under the assumption of a single causal variant per trait, including variants ±1 Mb surrounding the pQTL of interest. Associations were considered significant when the posterior probability H4 (PPH4; shared association with single causal variant) was ≥0.70 and suggestive for PPH4 > 0.50 (ref. ^89^). Associations with PPH4 < 0.50 were removed from further analyses. As a complementary approach, we used SuSiE colocalization^90^ (susieR package) to perform fine-mapping based on *z*-scores and linkage disequilibrium matrices using pQTLs in sample linkage disequilibrium matrix, allowing for the presence of multiple signals.

Discovery MR results were considered significant when passing the FDR Benjamini–Hochberg-corrected significance threshold (*P*_FDR_ < 0.05). In sensitivity analyses, we additionally corrected for the number of independent phenotypes tested, estimated using correlations between traits in the 3C-Dijon study applying the matrix spectral decomposition (matSpDlite^91^) method for WMH volume and each PVS location (*P*_FDR _< 1.2 × 10^−2^; 0.05/4).

#### Correlation of identified cSVD-associated proteins in plasma and CSF

##### Genetic correlation

Genetic correlation analyses were conducted using linkage disequilibrium score regression based on pQTL summary statistics. This approach aimed to (i) differentiate between shared genetic regulation and independent signals, (ii) assess biological coherence and clustering, and (iii) support the interpretation of 2SMR results. All proteins were tested; however, only those with sufficient single nucleotide polymorphism (SNP)-heritability estimates were retained for display, as low heritability values led to convergence issues. Genetic correlation could be reliably estimated for 24 proteins in CSF and 9 in plasma. *P* < 4 × 10^−5^ was used, correcting for the number of proteins tested and three situations: CSF-CSF, CSF-plasma and plasma-plasma; 0.05/(24 × 24) × 2 + (9 × 9)).

##### Inter-platform and intra-platform correlation

A subset of 259 ACE participants (Supplementary Methods) was analyzed in two independent experiments in CSF using the aptamer-based SomaScan 7K proteomic platform (SomaLogic). We considered the dataset with the adaptive normalization by maximum likelihood method for further analysis^10,11^. Additionally, another aliquot of these samples was analyzed in CSF with the antibody-based Olink Explore 3072 Panel measuring over 2,900 proteins (Olink Proteomics)^12^. Using this highly characterized sample, we conducted intra-platform (comparing SomaScan assays in CSF) and inter-platform (comparing SomaScan and Olink Explore in CSF) correlation analyses. We categorized proteomic measures in a single metric (1–9) accounting for reproducibility and reliability^92^. In another subset of 258 participants matched by draw date, protein-level correlations between CSF and plasma measured with SomaScan 7K at the same date were assessed.

### Follow-up of significant protein–cSVD associations

#### Cross-platform follow-up (direct protein measurements, Olink, plasma)

Protein–cSVD associations were followed up in two population-based cohorts with MRI phenotypes and plasma proteomics (Olink Explore 3072). In 3C-Dijon, 1,087 participants aged < 80 years (72.5 ± 4.1 years; 60.5% women) were included after quality control and exclusion of prevalent stroke/dementia; baseline plasma samples were profiled using proximity extension assay, following the manufacturer’s protocol^93^ at McGill Genome Center (Montreal, Canada), measuring 2,923 unique proteins. WMH volume was estimated from multimodal MRI (T1, T2, DP; 1.5T Siemens Magneton Scanner), and PVS burden in BG and WM was assessed using the SHIVA-PVS algorithm^94^ and a validated visual scale in HIP^95^. In the UK Biobank, 5,494 participants had Olink data (field ID: 1839) and brain MRI data (63.5 ± 7.9 years; 53.5% women); WMH volume and BG/WM PVS burden were estimated as in 3C-Dijon using T1-weighted images from the subset of participants with proteomics data (Supplementary Methods). All participants provided informed consent. Data preprocessing including plate-based normalization, and quality-control checks were conducted according to standardized Olink protocols.

**Extended Data Fig. 10:**
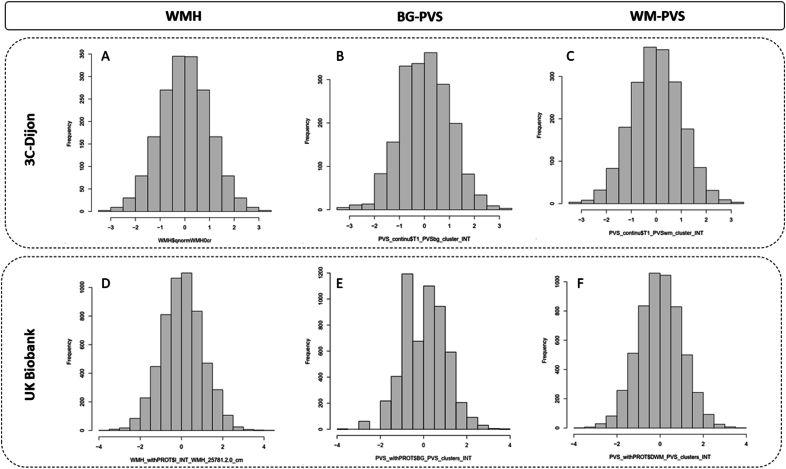
Distribution of MRI markers of cSVD in 3C-Dijon and UK-Biobank. Histogram of white matter hyperintensities (WMH) and perivascular spaces (PVS) distribution after normal inverse transformation in 3C-Dijon (**A**- **C**) and the UK Biobank (**D**-**F**).

We conducted linear and logistic regression of proteins with WMHs and PVSs adjusted for the delay between age at blood draw and age at the time of MRI, sex, batch effect, total intracranial volume (or mask volume for WMH in 3C-Dijon). WMHs and PVSs in BG and WM were inverse-normal transformed and PVSs in HIP values were dichotomized, comparing participants in the top quartile of PVS burden distribution to the rest, as previously described^5^. Data distribution was assumed to be normal but this was not formally tested. Individual data points are shown in Extended Data Fig. 10 to illustrate data distribution.

An inverse-variance weighted meta-analysis was performed using the metafor R package^96^. The heterogeneity of associations across studies was assessed using the Cochran–Mantel–Haenszel statistical test. Associations with *P* > 1.9×10^−3^ (0.05/26, correcting for 26 proteins available for follow-up) were considered. Significant associations were defined by *P*_FDR_ < 0.05. In addition, results of sensitivity analyses at *P*_FDR_ < 1.2 × 10^−2^ are displayed, accounting for the four phenotypes tested.

Associations of plasma protein levels with WMHs and PVSs were examined stratifying by hypertension status in 3C-Dijon and the UK Biobank, followed by meta-analysis. Hypertension was defined as SBP ≥ 140 mm Hg and diastolic blood pressure ≥ 90 mm Hg or use of antihypertensive medication (3C-Dijon: 235 hypertensive/852 non-hypertensive; UKB: 2,088/3,406). Direct analyses were further adjusted for SBP.

Protein–protein Spearman correlations were assessed in the UK Biobank using the corrplot R package, with significance at Bonferroni-corrected *P* < 7.7 × 10^−5^ (0.05/(26 × 26)−26).

#### Cross-ancestry follow-up (direct protein measurements, SomaScan, plasma)

A subset of 858 participants with brain imaging and plasma proteomic data from the Nagahama study, a prospective population-based cohort study initiated in 2007 in Nagahama, Japan (*N* = 10,082 at baseline, median age: 57.3 (41.6–64.7) years, 68% women), were used^97^ (Supplementary Methods). WMHs in Nagahama was generated using UBO detector^98^. PVS burden was estimated using the aforementioned machine-learning-based SHIVA-PVS algorithm^5,94^. Quality-control checks and proteomic measurement transformation (log2) were conducted according to standardized SomaScan protocols. After excluding participants for whom the estimation of the MRI marker was not possible, without proteomics measurements, with prevalent stroke, who had missing covariates or who had withdrawn their consent, a total of 785 participants were available for association analyses. We conducted linear regression for WMHs, WM-PVSs and BG-PVSs as continuous variables that were inverse-normal transformed and adjusted for age, sex, batch, total intracranial volume and the first four principal components. Associations at *P* < 0.05 were reported given the exploratory nature of these cross-ancestry analyses on a much smaller sample size.

#### Follow-up across the lifespan (pQTLs, SomaScan, plasma and CSF)

We conducted 2SMR analyses using the aforementioned pQTLs in plasma and CSF (instruments) and GWAS for WMHs and PVSs (outcomes). WMH and PVS GWAS were conducted in the i-Share study, an ongoing prospective population-based cohort study of French-speaking students^99^. We used a subsample of 1,748 participants aged 18–35 years, recruited in Bordeaux, France, for whom both brain MRI and genome-wide genotype data were available (mean age: 22.1 ± 2.3 years; 72.2% women)^100–102^. All participants provided informed consent, and MRi-Share participants received compensation of 40 euros. MRI protocol, genetic data quality-control and imputation procedures are detailed elsewhere^5,100–102^. For i-Share PVS GWAS summary statistics, we used previously described data^5^. For i-Share WMH GWAS summary statistics, we performed GWAS using the genome-wide linear mixed model implemented in REGENIE on WMH volume quantified using a recently developed algorithm^103^ (after excluding eight participants with multiple sclerosis or radiologically isolated syndrome)^104^. WMH volume was transformed using an indirect inverse-normal transformation (applying inverse-normal transformation to residuals from linear regression of WMHs adjusted for covariates (age at MRI, sex, total intracranial volume and the first four principal components of population stratification). These analyses were restricted to SNPs with an imputation score > 0.5 and a minor allele frequency > 0.01. Associations at *P* < 0.05 were reported given the exploratory nature of these cross-ancestry analyses on a much smaller sample size.

### Clinical significance

#### MR

Summary statistics for any (*N* = 73,652), ischemic (*N* = 62,100) and small vessel (*N* = 6,811) stroke were derived from the GIGASTROKE study incuding 1,234,808 controls^105^ and the largest GWAS for intracerebral hemorrhage (1,545 patients^106^). For dementia, we used summary statistics of the largest GWAS for Alzheimer’s disease comprising 71,880 Alzheimer’s disease cases^107^, including clinically diagnosed cases, and based on self-reported parental history as a proxy for diagnosis. We considered associations at *P* < 0.05 and reported significant findings at *P*_FDR_ < 0.05.

#### Observational survival analysis

We explored the relation of individual plasma protein levels (Olink Explore 3072) with incident stroke (any) and dementia (all-cause) in the UK Biobank and 3C-Dijon longitudinal population-based cohorts. After quality control and exclusion of prevalent stroke and dementia cases, *N* = 53,021 and *N* = 1,087 participants with plasma protein measurements were available in the UK Biobank and 3C-Dijon, of whom 1,400 and 1,471 developed stroke and dementia in the UK Biobank and 40 and 84 in 3C-Dijon, respectively. Event ascertainment in each cohort is detailed in Supplementary Methods. Cause-specific Cox models accounting for competing risk of death were used to explore associations with incident stroke and dementia, using age as a timescale, and adjusting for batch, sex and self-reported ancestry, and additionally educational attainment for associations with incident dementia. Analyses were then meta-analyzed using an inverse-variance weighted meta-analysis using the metafor R package^96^. The heterogeneity of associations across studies was assessed using the Cochran–Mantel–Haenszel statistical test, and associations with *P* > 2.1 × 10^−3^ (0.05/24, correcting for 24 proteins available for follow-up) were considered. Significant associations were defined by *P*_FDR_ < 0.05 and suggestive by *P* < 0.05.

We performed sensitivity analyses using a Fine–Gray subdistribution hazard model. These analyses were conducted in the UK Biobank, representing the largest contribution to the meta-analysis (*N* = 53,021), using the survival R package (version 3.8-3).

#### Associations with dementia subtypes

In the UK Biobank, dementia subtypes were defined using algorithmically determined outcomes (field: 42022), while in 3C-Dijon, they were based on clinical diagnoses over 12 years of follow-up. This resulted in 102 participants with vascular or mixed dementia and 376 with Alzheimer’s disease in 3C-Dijon, and 283 and 731 cases with vascular dementia and Alzheimer’s disease, respectively, in the UK Biobank. As described above, cause-specific Cox models were performed, adjusting for the same covariates, and subsequently meta-analyzed.

#### Cross-ancestry association analysis with stroke

We conducted 2SMR analyses in BBJ (first cohort study^108^), which recruited around 200,000 participants across 66 hospitals in Japan between 2003 and 2007. Proteomic profiling was conducted for a total of 2,886 individuals of East Asian ancestry from two previous studies^109,110^ with whole-genome sequencing datasets (Olink Explore 3072; mean age: 62.4 ± 14.5 years; 46.9% women). Data preprocessing and quality control were conducted according to standardized Olink protocols. Rank-based inverse-normal transformation was applied to protein-level measurements. pQTL summary statistics of serum protein levels were obtained for 19 available proteins (of the 49 cSVD-associated proteins from the discovery analysis) by meta-analyzing (using METAL^111^, inverse-variance weighted method; fixed-effect model) summary statistics generated in individuals from each study separately using REGENIE (v3.2.9)^104^ (adjusted for age, sex, age-squared, age × sex, age-squared × sex, batch and the first ten genotype principal components). Summary statistics of GWAS for ischemic (*N* = 17,493), large-artery atherosclerotic (*N* = 1,322), cardioembolic (*N* = 747) and small vessel (*N* = 4,876) stroke were obtained in the BBJ first cohort using REGENIE v3.2.9 (adjusted for age, sex and the first ten genotype principal components), excluding the samples used for proteomic profiling. Genotyping, quality control and imputation for BBJ samples used in the stroke GWAS were conducted as previously described^112^, except that the imputation was performed using a reference panel combining the 1000 Genome Project phase 3 v5 reference panel and 3,256 Japanese samples (JEWEL3k) samples^113^. Individuals without any type of stroke or cerebral aneurysm were used as controls. Instrument selection and MR were conducted following the methods previously described using a *P* threshold for clumping of 1 × 10^−6^ (Supplementary Methods).

### Biological interpretation

#### Pathway enrichment analysis

The GENE2FUNC analysis tool in FUMA (v1.5.4) was used to conduct gene-set enrichment analyses and detect significantly associated Gene Ontology biological processes^114^. GENE2FUNC uses a hypergeometric test to assess the overrepresentation of genes within predefined gene sets. The gene IDs used correspond to coding genes of identified proteins. We tested enrichment of the entire set of genes encoding cSVD-associated proteins identified in CSF and plasma, using the background set of genes encoding proteins tested for MR in each tissue, respectively. Benjamini–Hochberg multiple-testing correction was applied to these results (*P* < 0.05).

#### STEAP enrichment analysis

We performed a cell-type enrichment analysis using the STEAP tool (https://github.com/erwinerdem/STEAP/). This tool serves as an extension to CELLECT and integrates stratified LD score regression, MAGMA and H-MAGMA for enrichment analysis. pQTL summary statistics from the CSF and plasma datasets were preprocessed. Subsequently, expression specificity profiles were computed using single-cell RNA-seq data from human and mouse databases, including PsychENCODE DER-22, GSE67835, GSE101601, DroNc Human Hippocampus, Allen Brain Atlas MTG and LNG, Mousebrain, Tabula Muris, Descartes Human Cerebrum and Cerebellum. Cell-type enrichment analysis was conducted using MAGMA, H-MAGMA (which incorporates chromatin interaction profiles from human brain tissues in MAGMA) and stratified LD score regression. *P* values were Bonferroni-corrected for the number of independent cell types in each database.

#### Brain single-cell QTLs

Mapping of brain single-cell eQTLs was described elsewhere^115–117^. Briefly, single-nucleus RNA-seq libraries were prepared from dorsolateral prefrontal cortex (dPFC) of 424 participants from the ROSMAP cohort using the 10x Genomics Single Cell 3’ kit. Sequencing reads were processed and a unique molecular identifier count matrix was generated using Cell Ranger software (ver.6.0.0, 10x Genomics). Classification of cell types was performed by clustering cells by gene expression using the R package Seurat (ver. 4). A ‘Pseudobulk’ gene expression matrix was constructed by aggregating unique molecular identifier counts of the same cell type of the same donor and normalizing them to the log_2_ counts per million reads mapped values. Genotyping was performed by whole-genome sequencing and GATK. Mapping of *cis*-eQTLs was performed using Matrix-eQTL (ver. 2.3) for SNPs within 1 Mb of transcription start sites. Due to the sparsity of vascular cells in brain tissue, a specific dataset from ROSMAP using in silico vasculature enrichment was used for eQTL and expression analysis. Single-nucleus RNA-seq libraries were prepared from brain samples of 409 ROSMAP participants using the 10x Genomics Single Cell 3′ Kit (Supplementary Methods). Microglia states were defined from 152,459 microglial transcriptomes across 443 individuals (217 with Alzheimer’s disease and 226 controls) identifying 12 transcriptional states. Microglial nuclei were obtained from postmortem brain samples from the ROSMAP study across six brain regions (hippocampus, dPFC, mid-temporal cortex, angular gyrus, entorhinal cortex and thalamus). Using in silico sorting, 174,420 immune cells were collected from single-nucleus RNA-seq datasets using the STAR method forming 12 clusters of microglia. Those clusters were then defined as microglial states based on their molecular signature and function: MG0, hemostatic; MG1, neuronal surveillance; MG2, inflammatory I; MG3, ribosome biogenesis; MG4, lipid processing; MG5, phagocytic; MG6, stress signature; MG7, glycolytic; MG8, inflammatory II; MG10, inflammatory III; MG11, antiviral; MG12, cycling. Detailed methods regarding microglial state definitions are described elsewhere^35^.

### Proteomics-driven drug discovery

Using significant MR results from CSF and plasma, we restricted our analysis to drug-targeting proteins using four drug–gene databases (ChEMBL, pharmGKB, DrugBank and TTD). Following this methodology, eight drug-targeting proteins were identified for WMHs (EPO, LTF, TFPI, APOE, ARSB, CTSS, CTSB and EPHB4) and seven for PVSs (COL6A1, CTSB, GPNMB, PCSK9, FcRIIIA, heparin cofactor II, IL-6). Using public drug databases, we then curated drugs targeting those proteins in a direction compatible with a beneficial therapeutic effect against the corresponding cSVD phenotype based on MR estimates. The desired mode of action was defined as the opposite direction of the MR estimate. Once the drugs were identified, we searched the literature for a potential action of the drug.

### Statistics and reproducibility

No statistical methods were used to predetermine sample sizes, but our sample sizes are similar or larger to those reported in previous publications^12,13,32,77^. This study is based on summary statistics and observational cohort data. Randomization and blocking were not applicable as participants were not assigned to experimental groups. Data collection and preprocessing were not performed blind; however, the analyst was partially blinded to variable identities during analyses.

### Reporting summary

Further information on research design is available in the Nature Portfolio Reporting Summary linked to this article.

## Supplementary information

Supplementary Information Supplementary Methods, Figures and References.

Reporting Summary

Supplementary Tables Supplementary Table 1: List of available proteins after instrument selection for mendelian randomization in CSF and plasma pQTLs datasets. Supplementary Table 2: Association of the 46 cerebrospinal fluid-proteins associated with MRI-markers of cerebral small vessel disease using Mendelian randomization. Supplementary Table 3: Detailed results of cerebrospinal fluid cis-pQTL mendelian randomization with MRI-markers of cerebral small vessel disease, including sensitivity analyses: white matter hyperintensities (WMH) and perivascular spaces burden (PVS) in basal ganglia (BG), hippocampus (HIP) and white matter (WM). Supplementary Table 4: Association of the 9 plasma-proteins associated with MRI-markers of cerebral small vessel disease using Mendelian randomization. Supplementary Table 5: Detailed results of plasma cis-pQTL mendelian randomization with MRI-markers of cerebral small vessel disease, including sensitivity analyses (white matter hyperintensities, perivascular scpaces in white mater, hippocampus and basal ganglia). Supplementary Table 6: Association of the proteins associated with WMH and/or PVS with cerebral microbleeds using Mendelian randomization in CSF and plasma. Supplementary Table 7: Non-synonymous variants used as pQTLs for analysis. Supplementary Table 8: Multivariable MR using systolic blood pressure for each MRI-cSVD proteins identified in CSF in primary MR analysis. Supplementary Table 9: Cross-fluid follow-up of CSF protein-cSVD associations in plasma using Mendelian randomization. Supplementary Table 10: Cross-fluid follow-up of plasma protein-cSVD associations in CSF using Mendelian randomization. Supplementary Table 11: Description of the population with Olink Explore 3072 proteomic measurements in plasma in A. 3C-Dijon and B. UKBiobank. Supplementary Table 12: Cross-platform follow-up findings of cerebrospinal fluid and plasma proteins associated with white matter hyperintensities in an independent plasma dataset measured with Olink in A. The 3C cohort; B. The UK Biobank. Supplementary Table 13: Inverse variance weighted meta-analysis of protein levels measured with Olink with white matter intensities and perivascular spaces (PVS) in 3C cohort and the UK Biobank study. Supplementary Table 14: Correlation analysis of cSVD-associated proteins levels in CSF and plasma measured using Somascan 7K, and genetic correlations. Supplementary Table 15: Correlation p-values of protein levels measured in the UKB across the 26 cSVD-associated proteins using Spearman correlation (two-sided). Supplementary Table 16: Correlation analysis of cSVD-associated proteins levels in SomaScan and Olink in CSF (Methods) and plasma separately. Supplementary Table 17: Inverse variance weighted meta-analysis of protein levels measured with Olink with white matter intensities and perivascular spaces (PVS) in 3C cohort and the UK Biobank study A. stratified by hypertensive status focusing on WMH, B.on HIP-PVS exclusively using 3C-Dijon, C. adjusting for systolic blood pressure studying WMH, D studying HIP-PVS. Supplementary Table 18: Cross-ancestry follow-up of protein-cSVD associations using direct SomaScan measurements in the Nagahama study. Supplementary Table 19: Lifespan follow-up mendelian randomization of cerebrospinal fluid and plasma proteins associated with MRI markers of cerebral small vessel disease in older adults. Supplementary Table 20: Expression analysis of identified protein-cSVD associations using transcriptome wide association studies (GTEXv8 and single-cell RNA sequencing data from post-mortem dPFC) and mendelian randomization analyses. Supplementary Table 21: Mendelian randomization results testing the relation of MRI-cSVD associated CSF-proteins with stroke and dementia in European-ancestry populations. Supplementary Table 22: Mendelian randomization results testing the relation of MRI-cSVD associated plasma-proteins with stroke and dementia in European-ancestry populations. Supplementary Table 23: Inverse variance weighted meta-analysis of protein levels measured with Olink with stroke and dementia in the 3C Dijon cohort and the UK Biobank study. Supplementary Table 24: Effect of plasma protein level with the risk of A. all stroke and B. all cause dementia using Fine-Gray model in the UK Biobank (N=53,021). Supplementary Table 25: Proteins-cSVD association with dementia subtypes: metaanalysis of 3C-Dijon and UK Biobank with A. Alzheimer’s disease and B. Vascular or mixed dementia. Supplementary Table 26: Mendelian randomization results testing the relation of MRI-cSVD associated CSF-proteins with stroke in East-Asian-ancestry populations. Supplementary Table 27: Enrichment analysis of the coding-genes of the cSVD-proteins identified using cis-pQTL mendelian randomization using FUMA. A. Focusing on all identified cSVD-proteins (CSF&plasma). B. Focusing on proteins identified in CSF. Supplementary Table 28: Single-cell RNA sequencing datasets used in the single-cell type enrichment analysis (STEAP pipeline). Supplementary Table 29: Single-cell type enrichment analysis using STEAP. Supplementary Table 30: Mendelian randomization analysis using brain single cell sequencing data to explore association of genetically determined cell-type specific gene expression with MRI-markers of cerebral small vessel disease MRI-cSVD. Supplementary Table 31: Mendelian randomization analysis using brain single cell sequencing data enriched in vascular cells to explore association of genetically determined cell-type specific gene expression with MRI-markers of cerebral small vessel disease. Supplementary Table 32: Proteogenomics-driven Drug Discovery.

## Data Availability

We used publicly available data for analyses described in this paper, including data from the GWAS catalog (https://www.ebi.ac.uk/gwas/, study codes: GCST90244151, GCST011947, GCST007320, GCST90104539, GCST90162546), the DECODE project (https://www.decode.com/summarydata/), ChEMBL (https://www.ebi.ac.uk/chembl/), pharmGKB (https://www.pharmgkb.org/), DrugBank (https://go.drugbank.com/), TTD (https://db.idrblab.net/ttd/), CSF pQTL summary statistics available at NIAGADS (full summary statistics available to approved investigators through accession no. NG00130) and GWAS catalog (study code: GCST90421033–GCST90428040), NeuroGenomics and Informatics Center website (https://neurogenomics.wustl.edu/open-science/raw-data/) and ONTIME browser (https://ontime.wustl.edu/). Plasma proteomic data for the Knight ADRC participants are available at https://knightadrc.wustl.edu/professionals-clinicians/request-center-resources/. Requests for clinical or proteomic data from individual investigators will be reviewed to ensure compliance with patient confidentiality. For details on accessing available data and study protocols, see https://knightadrc.wustl.edu/.

## Code availability

We used publicly available tools from TwoSampleMR (v0.5.686, https://mrcieu.github.io/TwoSampleMR/), FUMA (v1.5.4, https://fuma.ctglab.nl/), coloc R package (v5.2.3, https://chr1swallace.github.io/coloc/), STRING (v12.0, https://string-db.org/), FUSION (v2.7.13, https://github.com/gusevlab/fusion_twas/), PLINK (v2.0, https://www.cog-genomics.org/plink/2.0/), OlinkAnalyze R package (v4.3.1, https://github.com/Olink-Proteomics/OlinkRPackage/), MungeSumstats R package (v1.16.0, https://github.com/neurogenomics/MungeSumstats/), ggplot2 R package (v3.5.2, https://github.com/tidyverse/ggplot2/), forestplot R package (v3.1.7, https://cran.r-project.org/web/packages/forestplot/), Betsholtz database (http://betsholtzlab.org/VascularSingleCells/database.html), metafor R package (v4.8-0, https://wviechtb.github.io/metafor/), SHIVA-PVS algorithm (https://github.com/pboutinaud/SHIVA_PVS/, T1.PVS/v1), STEAP (https://github.com/erwinerdem/STEAP/). Figures were created using ggplot2 R package (https://ggplot2.tidyverse.org/) and BioRender.com.
