# Supplementary material for: Proteogenomics in cerebrospinal fluid and plasma reveals new biological fingerprint of cerebral small vessel disease
Source: Nat Aging. Author manuscript; Available in PMC 2026 Feb 10. (PMC12705447; doi:10.1038/s43587-025-01006-w)
Supplement: 2 [file NIHMS2129453-supplement-2.pdf]

nature aging

Supplementary information
https://doi.org/10.1038/s43587-025-01006-w

# Proteogenomics in cerebrospinal fluid and plasma reveals new biological fingerprint of cerebral small vessel disease

In the format provided by the authors and unedited

# SUPPLEMENTARY APPENDIX

## Table of content

1. **Supplementary methods** ........ 2
2. **Supplementary Figures** ........ 7
3. **References** ........ 9

## 1. Supplementary methods

### Ethics committee

Study protocols were approved for all studies by the appropriate boards at their respective institutions:

- 3C-Dijon: Ethics committee of the University Hospital of Kremlin-Bicêtre
- iSHARE: Commission Nationale Informatique et Libertés (CNIL); CCTIRS (Comité Consultatif sur le Traitement de l'Information en matière de Recherche dans le domaine de la Santé); CPP (Comité de Protection des Personnes); l'Agence Nationale de Sécurité du Médicament et Hors Produit de Santé (ANSM)
- Nagahama : Ethics committee of Kyoto University Graduate School of Medicine and the Nagahama Municipal Review Board
- UK Biobank: National Research Ethics Service Committee North West-Haydock (reference 11/NW/0382)
- ACE: The study protocols are approved by the Clinical Research Ethics Commission of the Hospital Clinic (Barcelona, Spain)

### Three-City Dijon Study (3C-Dijon)

The 3C study is a research project conducted across three French cities (Bordeaux, Dijon, and Montpellier), involving a total of 9,294 participants. Its primary objective is to assess the risk of dementia and cognitive impairment associated with vascular factors. Eligibility criteria required participants to reside in the respective cities and be registered on the electoral rolls in 1999, be 65 years or older, and not be institutionalized. Approval for the study protocol was obtained from the Ethical Committee of the University Hospital of Kremlin-Bicêtre, and all participants provided informed consent. The data used in this project were collected as part of the 3C-Dijon study, which recruited 4,931 individuals between 1999 and 2001. Further details on the overall design of the 3C-Dijon study can be found elsewhere[1,2]. Participants aged less than 80 years and enrolled between June 1999 and September 2000 (n=2,763) were invited to undergo brain MRI scans. A total of 2,285 subjects agreed to participate (82.7% response rate), and 1,924 MRI scans were performed, with 120 non-interpretable. Consequently, cerebral white matter lesion measures were available for 1,800 participants. MRI scans were conducted using a 1.5-Tesla Magnetom scanner (Siemens, Erlangen, Germany). The T1- and T2-weighted images of each subject were initially aligned using the AIR package, followed by analysis using an optimized Voxel-Based Morphometry (VBM) protocol with Statistical Parametric Mapping 99 (SPM99), which was adapted to accommodate the structural characteristics of the aged brain. Automated image processing software was utilized to detect, measure, and localize white matter hyperintensities (WMH). Olink proteomic profiling, based on blood samples obtained at inclusion, was conducted in 1,100 participants with brain MRI measurements, without dementia at the time of the blood drawn,

**(Supplementary Table 22)** selected based on availability of brain MRI and amounts of plasma tubes left. Protein measurements were conducted on the Olink Explore 3072 panel using PEA technology, following the manufacturer's protocol[3], at McGill Genome Center (Montreal, Canada).

**Alzheimer Center Barcelona**

Ace Alzheimer Center Barcelona (ACE) was founded in 1995 and has collected and analyzed nearly 18000 genetic samples, diagnosed over 8000 patients, and participated in almost 150 clinical trials during its existence. For more details, visit www.fundacioace.com/en. In Ace, the syndromic diagnosis of all subjects was established by a multidisciplinary group of neurologists, neuropsychologists, and social workers. We assigned to healthy controls (HCs) including individuals with subjective cognitive decline (SCD) diagnosis a Clinical Dementia Rating (CDR) of 0, and mild cognitive impairment (MCI) individuals a CDR of 0.5. For the MCI diagnosis, we considered the classification of López *et al*, and Petersen's criteria[4–7]. We used the 2011 National Institute on Aging and Alzheimer's Association (NIA-AA) guidelines for AD diagnosis[5]. All Ace clinical protocols have been previously published[8–10]. We also obtained paired plasma and CSF samples[11] following consensus recommendations, and both biomaterials were stored at -80°C. The study protocols are approved by the Clinical Research Ethics Commission of the Hospital Clinic (Barcelona, Spain).

**Follow-up of significant protein-cSVD associations**

*Cross-platform follow-up*

Data were transformed and normalized to Olink's Normalized Protein eXpression (NPX) values, a relative protein quantification unit on a logarithmic base 2 scale. We generated PCA plots encompassing all proteins and samples, following which 3 samples were removed due to deviations greater than 5 standard deviations from the mean. Subsequently, separate PCAs were generated for each panel, leading to the exclusion of additional 9 samples with deviation greater than 5 SD from the mean totallin 1,088 participants after QC. Additionally, we removed 3 proteins for which over 50% of NPX values were below the protein's limit of detection (LOD) value. For the remaining proteins, NPX values falling below the LOD threshold were kept for analyses.

*PVS burden prediction in the UK Biobank*

We predicted the PVS burden in the brain with the SHIVA-PVS algorithm[12] (https://github.com/pboutinaud/SHIVA_PVS, T1.PVS/v1) applied on the T1-weigted (T1w) images from 5523 UKB subjects with both MRI with usable T1w images and proteomics data (there were 5826 subjects with both MRI and proteomics but T1w in 303 subjects were tagged as 'unusable' and excluded from the current analysis). We only quantified the PVS in T1w images acquired at the first time point for any subjects with repeated acquisitions.

A threshold of 0.5 (range [0 1]) has been applied to binarize the probability maps produced by the algorithm. The number of clusters, i.e. the number of PVSs in the deep white matter (DWM) and the basal ganglia (BG), was computed for each subject. Additionally, cluster size filter of 5 has been applied to the deep white matter (DWM), while no cluster size filter is applied to the basal ganglia (BG) quantification.

**Observationnal survival analysis.**

In UK Biobank, the earliest reported events for "any stroke" (field ID: 42006) and "all-cause dementia" (field ID: 42018) were used. Prevalent stroke and dementia cases were excluded (UK Biobank: Nstroke=920, Ndementia=50; 3C-Dijon: Nstroke=28, Ndementia=1) and analyses were conducted on incident cases based on the latest follow-up available (UK Biobank: Nstroke=1400, Ndementia=1471; 3C-Dijon: Nstroke=40, Ndementia=84).

**Dementia definition**

In the UKB, diagnoses of all-cause dementia and dementia subtypes (Alzheimer disease and vascular dementia) were obtained using hospital inpatient records from the Hospital Episode Statistics for England, the Scottish Morbidity Record data for Scotland, and the Patient Episode Database for Wales. ICD 9/10 codes mentioned in the table below, were used in the diagnosis of the dementia outcomes. Participants were asked if they had history of 'Dementia or Alzheimer's or Cognitive Impairment' to which they answered 'Yes' or 'No'. This code has been included in the list of 'All Cause Dementia'. From the baseline (2006-2010) period, the UK biobank participants were followed (2011-2014) up to the earliest incident dementia diagnosis, date of death, the last data collection date by the general practitioner, or the last time of hospital inpatient admission, whichever occurred first.

## Dementia code lists

| UK Biobank Self Report Codes | | | | | | |
|---|---|---|---|---|---|---|
| **Code Type** | **Code** | **Biobank Code Text** | **AD** | **VD** | **FTD** | **Dementia** |
| UK Biobank Self Report | Field 20002 Code 1263 | Dementia/Alzheimers/Cognitive Impairment | | | | ✓ |
| **ICD 9 Codes** | | | | | | |
| **Code Type** | **ICD 9 Code** | **ICD 9 Text** | **AD** | **VD** | **FTD** | **Dementia** |
| ICD 9 Code | 290.2 | Senile dementia, depressed or paranoid type | | | | ✓ |
| ICD 9 Code | 290.3 | Senile dementia with acute confusional state | | | | ✓ |
| ICD 9 Code | 290.4 | Arteriosclerotic dementia | | ✓ | | ✓ |
| ICD 9 Code | 291.2 | Other alcoholic dementia | | | | ✓ |
| ICD 9 Code | 294.1 | Dementia in other conditions classified elsewhere | | | | ✓ |
| ICD 9 Code | 331.0 | Alzheimer's disease | ✓ | | | ✓ |
| ICD 9 Code | 331.1 | Pick's disease | | | ✓ | ✓ |
| ICD 9 Code | 331.2 | Senile degeneration of brain | | | | ✓ |
| ICD 9 Code | 331.5 | Creutzfeldt-Jakob disease | | | | ✓ |
| **ICD 10 Codes** | | | | | | |
| **Code Type** | **ICD 10 Code** | **ICD 10 Text** | **AD** | **VD** | **FTD** | **Dementia** |
| ICD 10 Code | A81.0 | Sporadic Creutzfeldt-Jakob disease | | | | ✓ |
| ICD 10 Code | F00 | Dementia in Alzheimer's disease | ✓ | | | ✓ |
| ICD 10 Code | F00.0 | Dementia in Alzheimer's disease with early onset | ✓ | | | ✓ |
| ICD 10 Code | F00.1 | Dementia in Alzheimer's disease with late onset | ✓ | | | ✓ |
| ICD 10 Code | F00.2 | Dementia in Alzheimer's disease, atypical or mixed type | ✓ | | | ✓ |
| ICD 10 Code | F00.9 | Dementia in Alzheimer's disease, unspecified | ✓ | | | ✓ |
| ICD 10 Code | F01 | Vascular dementia | | ✓ | | ✓ |
| ICD 10 Code | F01.0 | Vascular dementia of acute onset | | ✓ | | ✓ |
| ICD 10 Code | F01.1 | Multi-infarct dementia | | ✓ | | ✓ |
| ICD 10 Code | F01.2 | Subcortical vascular dementia | | ✓ | | ✓ |
| ICD 10 Code | F01.3 | Mixed cortical and sub-cortical vascular dementia | | ✓ | | ✓ |

| | | | | | | |
|---|---|---|---|---|---|---|
| ICD 10 Code | F01.8 | Other vascular dementia | | ✓ | | ✓ |
| ICD 10 Code | F01.9 | Vascular dementia, unspecified | | ✓ | | ✓ |
| ICD 10 Code | F02 | Dementia in other diseases classified elsewhere | | | | ✓ |
| ICD 10 Code | F02.0 | Dementia in Picks disease | | | ✓ | ✓ |
| ICD 10 Code | F02.1 | Dementia in Creutzfeldt-Jacob disease | | | | ✓ |
| ICD 10 Code | F02.2 | Dementia in Huntington's disease | | | | ✓ |
| ICD 10 Code | F02.3 | Dementia in Parkinson's disease | | | | ✓ |
| ICD 10 Code | F02.4 | Dementia in HIV disease | | | | ✓ |
| ICD 10 Code | F02.8 | Dementia in other specified diseases classified elsewhere | | | | ✓ |
| ICD 10 Code | F03 | Unspecified dementia | | | | ✓ |
| ICD 10 Code | F05.1 | Delirium superimposed on dementia | | | | ✓ |
| ICD 10 Code | F10.6 | Mental and behavioural disorders due to use of alcohol - amnesic syndrome | | | | ✓ |
| ICD 10 Code | G30 | Alzheimer's disease | ✓ | | | ✓ |
| ICD 10 Code | G30.0 | Alzheimer's disease with early onset | ✓ | | | ✓ |
| ICD 10 Code | G30.1 | Alzheimer's disease with late onset | ✓ | | | ✓ |
| ICD 10 Code | G30.8 | Other Alzheimer's disease | ✓ | | | ✓ |
| ICD 10 Code | G30.9 | Alzheimer's disease unspecified | ✓ | | | ✓ |
| ICD 10 Code | G31.0 | Circumscribed brain atrophy | | | ✓ | ✓ |
| ICD 10 Code | G31.1 | Senile degeneration of brain | | | | ✓ |
| ICD 10 Code | G31.8 | Other specified degenerative diseases of nervous system | | | | ✓ |
| ICD 10 Code | I67.3 | Binswanger's disease | | ✓ | | |

***Acknowledgments:*** *Code list generated by Kathryn Bush, Tim Wilkinson, Christian Schnier, John Nolan and Cathie Sudlow on behalf of UK Biobank Outcome Adjudication Group.*

In the 3C-Dijon study, dementia was classified into AD, vascular dementia, and mixed dementia. The diagnosis of AD followed the criteria established by the National Institute of Neurological and Communicative Disorders and Stroke–Alzheimer's Disease and Related Disorders Association (NINCDS–ADRDA), while vascular dementia was diagnosed according to the National Institute of Neurological Disorders and Stroke–Association Internationale pour la Recherche et l'Enseignement en Neurosciences (NINDS–AIREN) guidelines[13,14]. Mixed dementia was defined as a diagnosis of AD combined with either cerebrovascular abnormalities visible on neuroimaging or a documented stroke history, along with significant executive dysfunction alongside an AD-like cognitive profile.

**Cross ancestry follow-up – Mendelian randomization pQTL (EAS) and subtypes of stroke.**

Brain imaging and plasma proteomic data from the Nagahama study, a prospective population-based cohort study initiated in 2007 in Nagahama, Japan (N=10,082 at baseline) were used[15]. Healthy participants aged 30 to 74 years were recruited between 2008 and 2010 from the general population of Nagahama (Japan) and followed-up 5 years after baseline between 2013 and 2015. Plasma proteomic measurements have been conducted on a subset of 2,000 individuals using Somascan 4.0. Of those, 858 had brain MRI measurements. All participants provided informed consent.

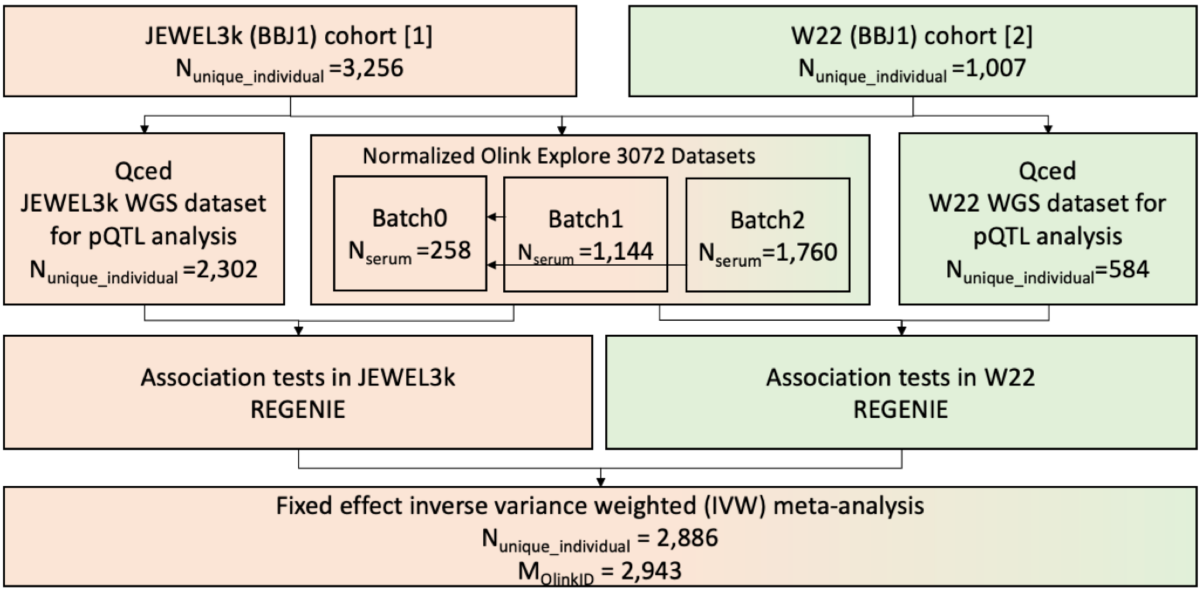


**Brain single cell expression quantitative trait loci (eQTL)**

Read counts were estimated using Cellranger 3.0.1 (10x Genomics) and the UMI count matrix was analysed using the Seurat R package v.3.2.0. Vascular enrichment was conducted in silico using cell sorting from post mortem human samples across seven different brain regions (prefrontal cortex, mid-temporal cortex, angular gyrus, entorhinal cortex, thalamus, hippocampus and mammillary body). Cell-type annotation was performed through clustering, annotating cell-type using a combination of canonical vasculature markers and whole-transcriptome cellular signatures. Detailed methods regarding sRNAseq and in silico vascular enrichment is described elsewhere[16,17].

## 2. Supplementary Figures

**Extended Data Figure 1. Discovery protein-cSVD associations in CSF and plasma using cis-pQTL mendelian randomization.** A. String plot of proteins associated with WMH. B. String plot of proteins associated with PVS (WM, BG and HIP). Network nodes represent proteins: colored nodes query proteins and first shell of interactors. Edges represent protein-protein associations. Cyan and pink edges are known interactions, cyan: from curated databases, and pink: experimentally determined. Green and

blue edges correspond to predicted interactions. Green: gene neighborhood, and blue: gene co-occurrence. Purple corresponds to protein homology, yellow to text mining and black to co-expression.

**Extended Data Figure 2. Multivariable Mendelian randomization.** Only proteins with >1 pQTL available for analysis are represented. Dots correspond to effect estimates (beta) and error bars to 95% confidence intervals. Forest plots correspond respectively to protein associations with WMH, WM-PVS, HIP-PVS and BG-PVS. Red lines correspond to primary MR results; blue lines correspond to multivariable MR (MVMR).

**Extended Data Figure 3. Genetic correlation between proteins associated with MRI-cSVD using protein quantitative trait loci.** A. Genetic correlation across pQTLs for 24 CSF protein levels associated with MRI-cSVD. B. Genetic correlation across pQTLs for 21 CSF or plasma proteins significantly associated with MRI-cSVD and available in both plasma and CSF (x axis: pQTL for CSF protein levels; y axis: pQTL for plasma protein levels). C. Genetic correlation across pQTLs for 9 plasma protein levels associated with MRI-cSVD. Genetic correlations were estimated using LD score regression. P-values are based on two-sided Wald tests of the null hypothesis, without adjustment for multiple comparisons. * $p<0.05$, ** $p<0.01$, *** $p<0.001$. Only proteins that converged for genetic correlation analyses are displayed. The exact p-values are reported in Supplementary Table 14.

**Extended Data Figure 4. Correlation of protein levels measured in the UKB across the 26 cSVD-associated proteins.** Correlations were estimated using Spearman correlation (two-sided). Unadjusted pvalues are displayed. *$p<7.7x10-3$ (Bonferroni corrected threshold). The exact p-values are reported in Supplementary Table 15.

**Extended Data Figure 5. Association of plasma protein levels with MRI-cSVD stratified on hypertension status.** Forest plots correspond to protein associations with WMH and HIP -PVS. Results corresponds to meta-analyses of association results in 3C-Dijon and UK Biobank (N=6,581; 2,088 hypertensive/3,406 non-hypertensive) for WMH; and 3C-Dijon only for HIP-PVS (N=1,087; 235 hypertensive/852 non-hypertensive), stratified on hypertension status: hypertensive (HTN), non-hypertensive (Non-HTN), all combined (Overall) and adjusted on systolic blood pressure (HTN adjusted). Dots represent effect estimates (beta for WMH and odds ratio for HIP-PVS) and error bars correspond to 95% confidence intervals.

**Extended Data Figure 6. Protein-cSVD associations with dementia subtypes (vascular and Alzheimer's disease): meta-analysis of 3C-Dijon and UK Biobank.** Results of cause-specific Cox models (Methods). N-vascular dementia=385; N-Alzheimer=1,107). Dots represent hazard ratios and errors bars corresponds to 95% confidence intervals.

**Extended Data Figure 7. Comparison of effect size estimates for MR associations of cSVD-associated proteins with ischemic stroke and small vessel stroke between Europeans (EUR) and East-Asians (EAS).** A. Ischemic Stroke. B. Small vessel Stroke.

**Extended Data Figure 8. Cell-type enrichment in single cell RNA-seq databases using STEAP.** Upset plot displays the number of significant enrichment results by protein (pQTL) horizontally and by cell-type vertically. CSF pQTLs are in black and plasma pQTLs are in blue. Details are displayed in Supplementary Table 25. Human and mouse single-cell databases are used in this analysis (Methods).

**Extended Data Figure 9. Single-nucleus gene expression/enrichment analyses.** A. Single-nucleus cerebrovascular gene expression data of cSVD-associated protein coding genes in dorsolateral prefrontal cortex (ROS-MAP study). B. Enrichment analyses of cSVD-associated protein coding genes in microglial states and vascular cells using in silico vascular enrichment (two-sided). Unadjusted pvalues are displayed. Dotted line corresponds to pval<0.05.

**Extended Data Figure 10. Histogram of white matter hyperintensities (WMH) and perivascular spaces (PVS) distribution after normal inverse transformation in 3C-Dijon (A- C) and the UK Biobank (D-F).**

## 3. References


1. Godin, O. *et al.* White matter lesions as a predictor of depression in the elderly: the 3C-Dijon study. *Biol. Psychiatry* **63**, 663–669 (2008).
2. 3C Study Group. Vascular factors and risk of dementia: design of the Three-City Study and baseline characteristics of the study population. *Neuroepidemiology* **22**, 316–325 (2003).
3. Lind, L. *et al.* Use of a proximity extension assay proteomics chip to discover new biomarkers for human atherosclerosis. *Atherosclerosis* **242**, 205–210 (2015).
4. Jessen, F. *et al.* A conceptual framework for research on subjective cognitive decline in preclinical Alzheimer's disease. *Alzheimers Dement. J. Alzheimers Assoc.* **10**, 844–852 (2014).
5. Lopez, O. L. *et al.* Risk factors for mild cognitive impairment in the Cardiovascular Health Study Cognition Study: part 2. *Arch. Neurol.* **60**, 1394–1399 (2003).
6. Petersen, R. C. *et al.* Mild cognitive impairment: a concept in evolution. *J. Intern. Med.* **275**, 214–228 (2014).

7. Petersen, R. C. *et al.* Mild cognitive impairment: clinical characterization and outcome. *Arch. Neurol.* **56**, 303–308 (1999).
8. Orellana, A. *et al.* Establishing In-House Cutoffs of CSF Alzheimer's Disease Biomarkers for the AT(N) Stratification of the Alzheimer Center Barcelona Cohort. *Int. J. Mol. Sci.* **23**, 6891 (2022).
9. Rodriguez-Gomez, O. *et al.* FACEHBI: A Prospective Study of Risk Factors, Biomarkers and Cognition in a Cohort of Individuals with Subjective Cognitive Decline. Study Rationale and Research Protocols. *J. Prev. Alzheimers Dis.* **4**, 100–108 (2017).
10. Moreno-Grau, S. *et al.* Genome-wide association analysis of dementia and its clinical endophenotypes reveal novel loci associated with Alzheimer's disease and three causality networks: The GR@ACE project. *Alzheimers Dement. J. Alzheimers Assoc.* **15**, 1333–1347 (2019).
11. Vanderstichele, H. *et al.* Standardization of preanalytical aspects of cerebrospinal fluid biomarker testing for Alzheimer's disease diagnosis: a consensus paper from the Alzheimer's Biomarkers Standardization Initiative. *Alzheimers Dement. J. Alzheimers Assoc.* **8**, 65–73 (2012).
12. Boutinaud, P. *et al.* 3D Segmentation of Perivascular Spaces on T1-Weighted 3 Tesla MR Images With a Convolutional Autoencoder and a U-Shaped Neural Network. *Front. Neuroinformatics* **15**, 641600 (2021).
13. Román, G. C. *et al.* Vascular dementia: Diagnostic criteria for research studies: Report of the NINDS-AIREN International Workshop*. *Neurology* **43**, 250–250 (1993).
14. McKhann, G. M. *et al.* The diagnosis of dementia due to Alzheimer's disease: recommendations from the National Institute on Aging-Alzheimer's Association workgroups on diagnostic guidelines for Alzheimer's disease. *Alzheimers Dement. J. Alzheimers Assoc.* **7**, 263–269 (2011).
15. Funada, S. *et al.* Longitudinal Analysis of Bidirectional Relationships between Nocturia and Depressive Symptoms: The Nagahama Study. *J. Urol.* **203**, 984–990 (2020).
16. Fujita, M. *et al. Cell-Subtype Specific Effects of Genetic Variation in the Aging and Alzheimer Cortex*. http://biorxiv.org/lookup/doi/10.1101/2022.11.07.515446 (2022) doi:10.1101/2022.11.07.515446.

17. Garcia, F. J. *et al.* Single-cell dissection of the human brain vasculature. *Nature* **603**, 893–899 (2022).
