# Supplementary material for: Proteogenomics in cerebrospinal fluid and plasma reveals new biological fingerprint of cerebral small vessel disease
Source: Nat Aging. 2025 Nov 20;5(12):2514–31. doi: 10.1038/s43587-025-01006-w (PMC12705447; doi:10.1038/s43587-025-01006-w)
Supplement: Supplementary file 1 [file 43587_2025_1006_MOESM2_ESM.pdf]

nature portfolio

Corresponding author(s): Stéphanie Debette & Carlos Cruchaga

Last updated by author(s): Aug 22, 2025

Reporting Summary

Nature Portfolio wishes to improve the reproducibility of the work that we publish. This form provides structure for consistency and transparency in reporting. For further information on Nature Portfolio policies, see our Editorial Policies and the Editorial Policy Checklist.

Statistics

For all statistical analyses, confirm that the following items are present in the figure legend, table legend, main text, or Methods section.

n/a	Confirmed	
☐	☒	The exact sample size (*n*) for each experimental group/condition, given as a discrete number and unit of measurement
☐	☒	A statement on whether measurements were taken from distinct samples or whether the same sample was measured repeatedly
☐	☒	The statistical test(s) used AND whether they are one- or two-sided<br>*Only common tests should be described solely by name; describe more complex techniques in the Methods section.*
☐	☒	A description of all covariates tested
☐	☒	A description of any assumptions or corrections, such as tests of normality and adjustment for multiple comparisons
☐	☒	A full description of the statistical parameters including central tendency (e.g. means) or other basic estimates (e.g. regression coefficient) AND variation (e.g. standard deviation) or associated estimates of uncertainty (e.g. confidence intervals)
☐	☒	For null hypothesis testing, the test statistic (e.g. *F*, *t*, *r*) with confidence intervals, effect sizes, degrees of freedom and *P* value noted<br>*Give P values as exact values whenever suitable.*
☒	☐	For Bayesian analysis, information on the choice of priors and Markov chain Monte Carlo settings
☒	☐	For hierarchical and complex designs, identification of the appropriate level for tests and full reporting of outcomes
☒	☐	Estimates of effect sizes (e.g. Cohen's *d*, Pearson's *r*), indicating how they were calculated

Our web collection on statistics for biologists contains articles on many of the points above.

Software and code

Policy information about availability of computer code

Data collection	No software was used for data collection
Data analysis	We used publicly available tools from TwoSampleMR (v0.5.686, https://mrcieu.github.io/TwoSampleMR/), FUMA (v1.5.4, https://fuma.ctglab.nl/), coloc R package (v5.2.3, https://chr1swallace.github.io/coloc/), STRING (v12.0, https://string-db.org/), FUSION (v2.7.13https://github.com/gusevlab/fusion_twas), PLINK (v2.0, https://www.cog-genomics.org/plink/2.0/), OlinkAnalyze R package (v4.3.1, https://github.com/Olink-Proteomics/OlinkRPackage), MungeSumstats R package (v1.16.0, https://github.com/neurogenomics/MungeSumstats), ggplot2 R package (v3.5.2, https://github.com/tidyverse/ggplot2), forestplot R package (v3.1.7, https://cran.r-project.org/web/packages/forestplot), Betsholtz database (http://betsholtzlab.org/VascularSingleCells/database.html), metafor R package (v4.8-0, https://wviechtb.github.io/metafor/), SHIVA-PVS algorithm (https://github.com/pboutinaud/SHIVA_PVS, T1.PVS/v1), STEAP (https://github.com/erwinerdem/STEAP/). Figures were created using ggplot2 R package (https://ggplot2.tidyverse.org/) and BioRender (https://app.biorender.com).

For manuscripts utilizing custom algorithms or software that are central to the research but not yet described in published literature, software must be made available to editors and reviewers. We strongly encourage code deposition in a community repository (e.g. GitHub). See the Nature Portfolio guidelines for submitting code & software for further information.

Data

Policy information about availability of data

All manuscripts must include a data availability statement. This statement should provide the following information, where applicable:

- Accession codes, unique identifiers, or web links for publicly available datasets
- A description of any restrictions on data availability
- For clinical datasets or third party data, please ensure that the statement adheres to our policy

We used publicly available data for analyses described in this manuscript, including data from GWAS catalog (https://www.ebi.ac.uk/gwas/, study code: GCST90244151, GCST011947, GCST007320, GCST90104539, GCST90162546), the DECODE project (https://www.decode.com/summarydata/), ChEMBL (https://www.ebi.ac.uk/chembl/), pharmGKB (https://www.pharmgkb.org/), DrugBank (https://go.drugbank.com/), TTD (https://db.idrblab.net/ttd/), CSF pQTL summary statistics available at NIAGADS (full summary statistics available to approved investigators through accession #NG00130), NeuroGenomics and Informatics Center website (https://neurogenomics.wustl.edu/open-science/raw-data/) and ONTIME browser (https://ontime.wustl.edu/). Plasma proteomic data for the Knight ADRC participants are available at https://knightadrc.wustl.edu/professionals-clinicians/request-center-resources/. Requests for clinical or proteomic data from individual investigators will be reviewed to ensure compliance with patient confidentiality. For details on accessing available data and study protocols, see https://knightadrc.wustl.edu.

Research involving human participants, their data, or biological material

Policy information about studies with human participants or human data. See also policy information about sex, gender (identity/presentation), and sexual orientation and race, ethnicity and racism.

Reporting on sex and gender	Our primary analyses rely on summary statistics used for mendelian randomization: all of the underlying GWAS included genetically determined sex as an adjustment variable. For individual-level data association analyses between protein levels and MRI or clinical outcomes, self-reported sex was used as an adjustement variable. Due to limited power (and unavailability of sex-specific GWAS summary statistics for our discovery analysis), we did not run sex-specific analyses in the individual-level follow-up.
Reporting on race, ethnicity, or other socially relevant groupings	Analyses were conducted using GWAS summary statistics of European and East-Asian ancestry participants separately; ancestries were genetically inferred using principal components analyses. Individual-level analyses were also conducted separately in European and East-Asian ancestry groups, equally defined using genetically inferred principal components, and additionally adjusted for principal components of population stratification.
Population characteristics	For Mendelian Randomization, protein quantitative trait loci were used as exposure and obtained from up to 35,559 participants of Europeans ancestry; GWAS summary statistics of WMH (N=48,454) and PVS (N up to 40,025) were used as outcome. Further exploration in young adults (22.1±2.3 years, 72.2% women, N=1,748) were used for GWAS of WMH and PVS and used as outcome in the MR. Further explorations using individual-level datasets leveraged the 3C-Dijon cohort (72.5 ±4.1 years, 60.5% women, N=1,043), the UK Biobank (63.5±7.9 years, 53.5% women, N=5,494) and the Nagahama (68.3±5.3 years, 64.4% women, N=785). For individual-level analyses, ancestry-specific linear or logistic regression have been performed, adjusting for the delay between age at blood draw and age at the time of MRI, sex, intracranial volume (for WMH), relevant principal components of population stratification, and batch effect of proteomic measurements.Further analysis were startified based on hypertension status. Detailled population characteristics by study and phenotype definitions can be found in the Methods and Supplementary Methods sections and in Supplementary Table 12.
Recruitment	All participating studies had population-based recruitment strategies. Details can be found in the Methods section and Supplementary Methods.
Ethics oversight	Study protocols were approved for all studies by the appropriate boards at their respective institutions, these have been reported in the Supplementary Methods. Study protocols were approved for all studies by the appropriate boards at their respective institutions:<br>- 3C-Dijon: Ethics committee of the University Hospital of Kremlin-Bicêtre<br>- iSHARE: Commission Nationale Informatique et Libertés (CNIL); CCTIRS (Comité Consultatif sur le Traitement de l'Information en matière de Recherche dans le domaine de la Santé); CPP (Comité de Protection des Personnes); l'Agence Nationale de Sécurité du Médicament et Hors Produit de Santé (ANSM)<br>- Nagahama : Ethics committee of Kyoto University Graduate School of Medicine and the Nagahama Municipal Review Board<br>- UK Biobank: National Research Ethics Service Committee North West-Haydock (reference 11/NW/0382)<br>- ACE: The study protocols are approved by the Clinical Research Ethics Commission of the Hospital Clinic (Barcelona, Spain)

Note that full information on the approval of the study protocol must also be provided in the manuscript.

Field-specific reporting

Please select the one below that is the best fit for your research. If you are not sure, read the appropriate sections before making your selection.

☒ Life sciences ☐ Behavioural & social sciences ☐ Ecological, evolutionary & environmental sciences

Life sciences study design

All studies must disclose on these points even when the disclosure is negative.

Sample size	We conducted a multi-pronged approach to assess the association of protein from CSF and plasma with MRI markers of cerebral small vessel disease: WMH and PVS. First, we conducted a Mendelian Randomization using the largest and unique CSF pQTL dataset (N=3,107; measuring 7,028 proteins) and the largest plasma pQTL dataset (N=35,559, measuring 4,907 proteins) as exposure; and the largest GWAS meta-analyses of WMH (N=48,454) and extensive PVS in its 3 sublocations (white matter, basal ganglia, hippocampus) (N up to 38,525) as outcome. Those datasets were of Europeans ancestry only. As stated in the Methods section "No statistical methods were used to pre-determine sample sizes but our sample sizes are similar or larger to those reported in previous publications13,81,83,84."
Data exclusions	Participants were excluded from analysis if they had co-morbidities modifying the measurement of the phenotype (prevalent stroke, prevalent dementia, multiple sclerosis or radiologically isolated syndrome), missing covariates or withdrawn consent. Proteins were excluded if they failed quality control filters decribed in the Supplementary Methods (page 13).
Replication	We conducted multiple follow-up analyses, across ancestries and across the lifespan, in the iSHARE cohort (N=1,748), the 3C-Dijon cohort (N=1,087), the UK Biobank (N=5,794) and the Nagahama cohort (N=785). These are described in detail in the Method section (pages 40-44) and Figure 1.
Randomization	We did not allocate participants into experimental groups. However we conducted Mendelian randomization analyses based on genetic associations in the study population (described in the Methods). As stated in Methods section: "This study is based on summary statistics and observational cohort data. Randomization and blocking were not applicable as participants were not assigned to experimental groups."
Blinding	Not relevant for the present study design (strictly speaking MRI measurements of WMH and PVS burden were measured blinded to the protein levels and vice versa). MR was conducted using summary statistics of available datasets, not individual level data.

Reporting for specific materials, systems and methods

We require information from authors about some types of materials, experimental systems and methods used in many studies. Here, indicate whether each material, system or method listed is relevant to your study. If you are not sure if a list item applies to your research, read the appropriate section before selecting a response.

Materials & experimental systems

n/a	Involved in the study	
☒	☐	Antibodies
☒	☐	Eukaryotic cell lines
☒	☐	Palaeontology and archaeology
☒	☐	Animals and other organisms
☒	☐	Clinical data
☒	☐	Dual use research of concern
☒	☐	Plants

Methods

n/a	Involved in the study	
☒	☐	ChIP-seq
☒	☐	Flow cytometry
☐	☒	MRI-based neuroimaging

Plants

Seed stocks	NA.
Novel plant genotypes	NA.
Authentication	NA.

Magnetic resonance imaging

Experimental design

Design type	Population-based brain imaging study.

Design specifications	The recruitment strategy for each cohort studies (3C-Dijon, iSHARE, Nagahama and UK Biobank) is described in the Methods section.
Behavioral performance measures	There are no behavioral performance measures in this study.

Acquisition

Imaging type(s)	Brain MRI with T1-, T2-weighted, FLAIR and proton density sequences images. Study-specific information is described in the Method and Supplementary Methods section.
Field strength	1.5 and 3.0 Tesla.
Sequence & imaging parameters	Study-specific sequence and imaging parameters informations is described in Methods section (page 40-44).
Area of acquisition	Brain.

Diffusion MRI ☐ Used ☒ Not used

Preprocessing

Preprocessing software	Study-specific information is described in the Methods section (page 17-23)
Normalization	Study-specific information is described in the Methods section (page 17-23)
Normalization template	Study-specific information is described in the Methods section (page 17-23)
Noise and artifact removal	Study-specific information is described in the Methods section (page 17-23)
Volume censoring	Study-specific information is described in the Methods section (page 17-23)

Statistical modeling & inference

Model type and settings	We conducted multivariable linear and logistic regression of individual proteins with WMH volume and PVS burden adjusted for the delay between age at blood draw and age at the time of MRI, sex, batch effect, total intracranial volume (or mask volume for WMH in 3C-Dijon). WMH volume and PVS burden in basal ganglia and white matter were inverse normal transformed and PVS in hippocampus values were dichotomized, comparing participants in the top quartile of PVS burden distribution to the rest.<br>Cause-sepcific cox models and Fine-Gray models were used to assess the association of protein levels with incident stroke and dementia in the UK Biobank and 3C-Dijon using age as a time scale, and adjusting for batch, sex and self-reported ancestry, and additionally educational attainment for associations with incident dementia.
Effect(s) tested	Association of protein levels in whole blood with white matter hyperintensities and perivascular spaces burden in white matter, basal ganglia and hippocampus.

Specify type of analysis: ☒ Whole brain ☐ ROI-based ☐ Both

Statistic type for inference<br>(See Eklund et al. 2016)	Non relevant for this study.
Correction	False discovery rate Benjamini Hochberg 5%.

Models & analysis

n/a	Involved in the study
☒	☐ Functional and/or effective connectivity
☒	☐ Graph analysis
☒	☐ Multivariate modeling or predictive analysis
